# Harnessing Machine Learning to Uncover Hidden Patterns in Azole-Resistant CYP51/ERG11 Proteins

**DOI:** 10.3390/microorganisms12081525

**Published:** 2024-07-25

**Authors:** Otávio Guilherme Gonçalves de Almeida, Marcia Regina von Zeska Kress

**Affiliations:** Faculdade de Ciências Farmacêuticas de Ribeirao Preto, Universidade de São Paulo, Ribeirão Preto 14040-903, SP, Brazil; otavio.almeida@usp.br

**Keywords:** CYP51, ERG11, machine learning, azoles, fungal resistance

## Abstract

Fungal resistance is a public health concern due to the limited availability of antifungal resources and the complexities associated with treating persistent fungal infections. Azoles are thus far the primary line of defense against fungi. Specifically, azoles inhibit the conversion of lanosterol to ergosterol, producing defective sterols and impairing fluidity in fungal plasmatic membranes. Studies on azole resistance have emphasized specific point mutations in CYP51/ERG11 proteins linked to resistance. Although very insightful, the traditional approach to studying azole resistance is time-consuming and prone to errors during meticulous alignment evaluation. It relies on a reference-based method using a specific protein sequence obtained from a wild-type (WT) phenotype. Therefore, this study introduces a machine learning (ML)-based approach utilizing molecular descriptors representing the physiochemical attributes of CYP51/ERG11 protein isoforms. This approach aims to unravel hidden patterns associated with azole resistance. The results highlight that descriptors related to amino acid composition and their combination of hydrophobicity and hydrophilicity effectively explain the slight differences between the resistant non-wild-type (NWT) and WT (nonresistant) protein sequences. This study underscores the potential of ML to unravel nuanced patterns in CYP51/ERG11 sequences, providing valuable molecular signatures that could inform future endeavors in drug development and computational screening of resistant and nonresistant fungal lineages.

## 1. Introduction

Fungal resistance to antifungals is a public health concern because limited resources are available for the treatment of mycoses [1]. In light of the increasing risk of fungal infections and the growing challenges associated with resistance and treatability, the World Health Organization has issued the inaugural list of priority fungal pathogens. This list serves as a guide for research, development, and public health initiatives, aiming to enhance knowledge acquisition and foster global comprehension and response to fungal-related concerns, among other critical objectives [2]. Much is known about the mechanisms of resistance to azoles, which is considered a multifactorial process [1,3] related not only to a single gene but also to efflux pumps, metabolic activity of the fungus, differential ergosterol compositionality, and mechanisms related to stress response, such as chaperone gene expression [3]. Comprehending this intricate multifactorial process proves challenging in laboratory settings. Many experiments involving the deletion of crucial genes involved in metabolic pathways are required to assess their role in resistance and the integration of multiple cross-related mechanisms from a dynamic perspective. Therefore, the study of single-gene-coding effectors recognized as a part of the resistance-mediated process remains persistent in the literature [1,4].

Among the extensively studied effectors of azole resistance are the CYP51 and ERG11 genes in filamentous fungi and yeast, respectively. These are homologous genes and are interchangeably treated as synonymous genes. Azoles act on membrane integrity by interfering with 14α-demethylases, also known as CYP51p or ERG11p, which are members of the cytochrome P450 monooxygenase superfamily [5,6]. These enzymes catalyze a crucial step in the biosynthesis of ergosterol. The inhibition of ergosterol biosynthesis is known to result in the accumulation of ergosterol precursors in the cell membrane, causing alterations in its fluidity and permeability and ultimately affecting the overall membrane structure. Additionally, the accumulation of deleterious sterols impairs membrane-bound enzymes, including chitin-synthase, and enzymes involved in detoxifying reactive oxygen species [5,6].

Several studies have focused on comparing CYP51 and ERG11 gene and protein sequences through global alignments to unravel patterns of conserved regions and their potential relevance to azole resistance. The study by [7] evaluated and furnished a comprehensive review evaluating the presence of amino acid substitutions in CYP51 sequences of *Aspergillus fumigatus* and the association between those substitutions and the resistance susceptibility profile. Similar approaches have been described for *Candida albicans* [8], Mucormycetes [9], the *Fusarium solani* species complex [10], and various other fungi [11,12].

Recent advancements in artificial intelligence (AI), particularly in machine learning (ML), have significantly enhanced the performance of data-driven applications across diverse domains. The field of deep neural networks and deep learning has played a crucial role in this progress. Recently, there has been a growing prevalence of ML models and advanced deep learning methods in the health sciences domain [13]. This increasing significance can be attributed to the remarkable progress in ML and the development of data-driven products. The availability of extensive structured and unstructured data, especially clinical and experimental data, has further fueled this trend [14]. In particular, the health field has experienced substantial benefits from adopting AI-driven solutions [15]. These advancements have been instrumental in various aspects of clinical decision-making and the management of infectious diseases [16]. Although there have been promising outcomes in hospital settings [17,18], antibiotic prescribing and management are exceptions. Beyond traditional stewardship programs, the importance of ML and deep learning has risen notably in addressing the antimicrobial resistance (AMR) challenge [19]. There is a call for continued investigation in this field to take advantage of recent advancements in ML and deep learning for a more robust approach to tackling the issue of AMR. Today, ML is a powerful tool for identifying hidden patterns in complex datasets and is now a reality in microbiology [20].

The random forest algorithm is a supervised learning algorithm [21] that requires training data to discern patterns and determine parameters. These parameters, numerical values derived from the data, constitute the foundation of a mathematical model. The optimization process involves varying numeric values, such as those related to random forest depth and sample splitting, through a grid search. This ensures the identification of optimal hyperparameters, improving model performance. Regarding supervised learning, splitting the dataset into training and test data is convenient. Typically, the training data comprise 20% to 30% of the original dataset, while the test data constitute 70% to 80%. The latter is used for model evaluation using dedicated metrics (e.g., accuracy, precision, and recall). To prepare numerical data from biological sequences such as DNA, RNA, or proteins, analysts must process these sequences to obtain significant numerical values reflecting various biological properties of the molecules [22].

A series of molecular descriptors can numerically represent protein sequences, most of which refer to physiochemical properties such as hydrophobicity, the composition of amino acids, and their relative frequencies in one-, di-, and tri-amino acids, or polarity, isoelectric point, probability of substitution using BLOSUM matrices, and others. This numerical description facilitates the comparison of diverse characteristics of biological sequences, extending beyond their structural aspects [23]. The resulting data can be summarized into a feature matrix suitable for supervised learning purposes.

ML methods employ algorithms to learn and predict AMR phenotypes directly from patients’ clinical, demographic, and living-condition data [19]. Thus, this approach has been extended to sequenced bacterial genomes [24] and adapted to MALDI-TOF MS data, enabling the detection of antifungal resistance in species such as *C. albicans* and *Aspergillus flavus* [25,26], among other applications.

In the context of fungal CYP51/ERG11 protein sequences, which were characterized by a notable degree of conservation among fungi, employing ML to identify novel attributes in these sequences is valuable. This approach can help address inquiries such as “What physiochemical properties in CYP51/ERG11 proteins might partially account for azole resistance in fungal strains?”. These questions pose a challenge when relying solely on visual inspection of multiple global alignments of protein sequences. In addition, the hotspot-based approach in ML, which involves the presence of determined amino acids in conserved regions of alignments, lacks the mathematical background to assess differences among these conserved sequences quantitatively. The differentiation among these proteins can be quantified using protein descriptors, which are configured to mathematically describe and evaluate the molecular characteristics of proteins. These descriptors encompass numerical values summarizing various molecular properties, including charge, molecular weight, isoelectric point, polarity, hydrophobicity, frequency of amino acids, or values derived from algorithmic techniques reconstructing n-dimensional structures (i.e., 2-D or 3-D structures) of proteins [27]. Thus, descriptors have become valuable tools for effectively representing the molecular characteristics of CYP51 and ERG11 protein sequences in a manner conducive to the use of ML algorithms for extracting patterns and learning from the data. This approach goes beyond properties related to the global alignment of proteins for hotspot identification or the calculation of identity and similarity among closely related sequences.

This study aimed to unravel hidden patterns related to physiochemical descriptors of the CYP51/ERG11 proteins utilizing supervised learning ML algorithms. These patterns could provide insights into often overlooked resistance-related properties within protein sequences obtained from susceptible (wild-type phenotypes) and resistant (non-wild-type phenotypes) fungal strains. The information obtained from the data aims to enhance our comprehension of the functional disparities between a protein derived from a WT strain and one derived from an NWT strain. Additionally, the model developed in this study could be employed to characterize CYP51/ERG11 proteins and their isoforms, particularly for screening purposes.

## 2. Materials and Methods

### 2.1. CYP51 and ERG11 Amino Acid Sequences

Protein sequences of fungal CYP51 and ERG11 were selected from the National Center for Biotechnology (NCBI) protein database (Protein [Internet]. Bethesda (MD): National Library of Medicine (US), National Center for Biotechnology Information; 2004—[cited 1 August 2023]. Available from: https://www.ncbi.nlm.nih.gov/protein/). The search focused on publications describing CYP51/ERG11 DNA sequences deposited on NCBI and associated MICs. The criteria for selection were: (i) sequences derived from Sanger DNA sequencing and documented in published papers, and (ii) provides minimum inhibitory concentration (MIC) data linked to the originating sequence isolates, excluding *Fusarium* spp. (also referred to as *Neocosmospora* spp. by some authors) [28]. The accession numbers for the predicted proteins were used to batch-download the protein sequences using the Entrez direct command line tool. In total, 282 protein sequences with associated MIC values were successfully obtained.

**Figure 1:**
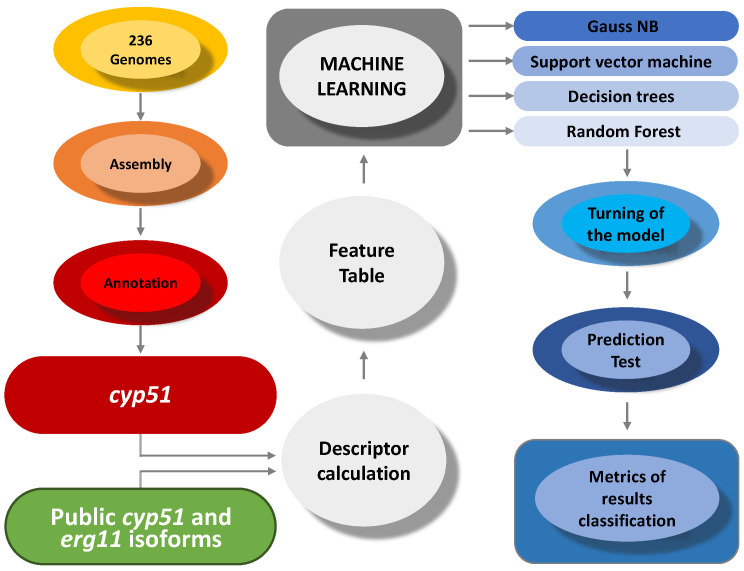
Experimental design for CYP51/ERG11 isoforms obtained from public databases and machine learning modeling.

In addition, all MIC data were revisited and re-evaluated to ensure adherence to standards and achieve precise sequence categorization into wild-type (WT) and non-wild-type (NWT) epidemiological cutoff values (ECVs) criteria. This process followed the standard reference values described in the CLSI and EUCAST protocols to avoid misestimations in subsequent analyses. The ECVs established by Espinel-Ingroff et al. (2016) [29] were used for *Fusarium* spp. (*Neocosmospora* spp.) as the reference values for classifying protein sequences as WT or NWT. For *Candida* spp. and *Aspergillus* spp., the ECV was according to protocol M59, CLSI [30]. Additionally, two publications [31,32] containing *Aspergillus fumigatus* whole-genome sequences were selected. These studies recorded NWT and WT genomes based on single-nucleotide polymorphism and MIC surveys. Subsequently, raw reads from 236 genomes (see Supplementary Table S1) were downloaded from the BioProject database of NCBI (https://www.ncbi.nlm.nih.gov/bioproject/; accessed on 1 March 2023) using accession numbers referenced in the selected publications. The reads underwent quality filtering with the bbduk tool (http://sourceforge.net/projects/bbmap/, accessed on 15 March 2023) using the parameters “hdist=1 tpe tbo qtrim=rl trimq=30 maq=30 minlen=90” and were assembled using Spades v3.13.1 [33] with the parameter “--careful”. A custom database of CYP51, including its isoforms and ERG11 protein sequences, was built using DIAMOND v2.0.7 [34], utilizing the 282 protein sequences retrieved previously. Subsequently, gene and protein prediction for the assembled genomes was performed using the Funannotate tool v1.8.15 with parameters “--species “Aspergillus””, and “--busco_seed_species aspergillus_fumigatus”, which incorporate the tools Augustus 3.5.0 [35] and Genemark [36] for ab initio and supervised learning predictions, respectively. The predicted proteins were then subjected to a DIAMOND [34] BLASTP algorithm against the custom database, with the best matches (evalue ≤ 0.001 and maximum identity) filtered for CYP51 isoform identification in the predicted proteins of the genomes. Finally, CYP51 sequences were obtained using the seqtk tool (https://github.com/lh3/seqtk; accessed on 1 March 2023) with the parameter “subseq” to retrieve the respective proteins annotated via blasting from the FASTA files. The resulting proteins (n = 472) and their metadata were combined with the publicly available proteins retrieved from NCBI into a single FASTA file to construct the feature table for ML purposes. The experimental design is summarized in Figure 1.

In terms of representativeness, the dataset encompasses sequences from various fungal species: *Aspergillus awamori* (n = 11; 11 WT), *A. flavus* (n = 63; 36 WT and 27 NWT), *A. fumigatus* (n = 538; 211 WT and 327 NWT), *A. lucuhensis* (n = 2; 2 WT), *A. niger* (n = 11; 10 WT and 1 NWT), *A. tubingensis* (n = 12; 12 WT), *Candida glabrata* (n = 23; 8 WT and 15 NWT), *C. krusei* (n = 4; 1 WT and 3 NWT), *C. parapsilopsis* (n = 18; 1 WT and 17 NWT), *C. tropicalis* (n = 51; 22 WT and 29 NWT), *Fusarium keratoplasticum* (n = 15; 15 NWT), *Neocosmospora falciformis* (n = 2; 2 NWT), and *N. suttoniana* (n = 4; 4 NWT). The accession numbers for the CYP51 and ERG11 genes along with their cognate proteins and associated metadata are summarized in Supplementary Table S1.

### 2.2. Sequences Processing and Descriptors Calculations

The protein sequences were evaluated in terms of quality to exclude those presenting unrecognizable amino acids, such as the “X” character, which corresponds to ambiguous base calls (“N”) in sequenced DNA bases. To achieve this goal, a custom script, check_aa.py, was used to identify and exclude sequences with “X” characters, resulting in a refined FASTA file suitable for subsequent analyses. After processing spurious sequences, 340 WT and 409 NWT protein sequences were selected for further analysis (Supplementary Table S1).

The proteins’ descriptors were calculated using the R package 2.30.1 protr [37] and a Python 3.10.10 package written originally in R named peptides [38]. Various descriptors were calculated using the protr R package, including amino acid composition (AAC), dipeptide composition (DC), tripeptide composition (TC), composition (CTDC), transition (CTDT), and distribution (CTDD) of encoded classes in the protein amino acid sequences, as well as pseudo-amino acid composition (PAAC) and amphiphilic pseudo-amino acid composition (APAAC). Additionally, using the peptide python 3.10.10 package, several quantitative structure–activity relationship (QSAR) descriptors were calculated, such as BLOSUM indices, Cruciani properties, FASGAI vectors, Kidera factors, MS-WHIM scores, PCP descriptors, ProtFP descriptors, Sneath vectors, ST-scales, T-scales, VHSE-scales, and Z-scales.

### 2.3. Feature Table Building and Supervised Machine Learning Analysis

Supervised ML was employed in this study to classify CYP51 and ERG11 protein sequences into WT and NWT groups based on chemical signatures within the sequences. The feature table was constructed by performing a total joint of two tables generated from processing protein sequences using selected tools for descriptors calculations, all within a Google Colab notebook. For the ML analysis, the Scikit-learn library [39] and its methods were used in the Colab environment.

The following steps were taken to build a model employing four supervised ML algorithms (random forest, support vector machines (SVM), decision trees, and GaussNB). First, the feature table was split into a training set (30% of the data) and a test set (70% of the data). Then, various data normalization methods (StandardScaler, MinMaxScaler, RobustScaler, QuantileTransformer with the options “normal” and “uniform”, and Normalizer) were evaluated for each ML algorithm on the training dataset to determine the most effective scaling method. Following the selection of the best scaling method for each algorithm based on their performance to achieve the highest accuracy, four learning models (one for each ML algorithm) were built and evaluated using five rounds of cross-validation in terms of accuracy, using the receiver operating characteristics and area under the curve (ROC–AUC) method and Matthews correlation coefficient (MCC score). Additionally, the algorithms were also compared in terms of accuracy on both the training and test datasets, and the algorithm exhibiting the best performance in both sets was selected for further model optimization.

### 2.4. Model Optimization and Evaluation

The optimal model, exhibiting the best performance, was generated using the random forest algorithm, as detailed in the upcoming Section 3. To further optimize this model, a grid search with cross-validation (GridSearchCV method) was performed by varying the following hyperparameters: “max_depth” (None, 2, 4, 8, 10, 12), “min_samples_split” (2, 5, 10), “criterion” (gini, entropy, log_loss), “max_features” (sqrt, log2, None), and “bootstrap” (False, True). The chosen scaling method for this optimization process was StandardScaler. After optimization, the evaluation of the model was performed using a classification report matrix. This matrix, displaying metrics such as accuracy, precision, recall, and f1 score, was plotted using the Seaborn library in Python. The metrics were derived by comparing the predictions between the training and test datasets. Moreover, a confusion matrix was plotted using the matplotlib library in Python, facilitating a comparison of false-positive, false-negative, true-positive, and true-negative predictions based on the labels WT and NWT.

### 2.5. Feature Importance Identification and Permutation Importance Analysis

Feature importance was determined via the random forest algorithm during the learning process. The top 20 most important features of the learning process are summarized in a bar plot report, highlighting their mean decrease in impurity (MDI) metric. To compute the weight of each feature in terms of accuracy for both the training and test datasets, a permutation importance analysis was conducted through five repetitions utilizing the permutation_importance method from the Sklearn (v1.0) Python (v3.10.10) package for machine learning. However, it is worth noting that permutation analysis tends to overestimate the importance of continuous or high-cardinality categorical variables [40]. To address this issue, the entire dataset was processed before the permutation analysis. Initially, Spearman correlation was performed to detect highly correlated features (>95%) for removal, reducing the dataset dimensionality. This processing reduced the original feature table of 8772 attributes to 6184 features. Subsequently, the random forest model, utilizing the previously optimized parameters, was constructed to compute the importance of the feature. Boxplots were plotted to show the variation ranges of each feature’s importance using the matplotlib library.

### 2.6. Global Alignment and Logo Construction

Given the homologous nature and evolutionary conservation of the CYP51 and ERG11 protein sequences [6,41,42], a comprehensive alignment of all 749 protein sequences was built using Clustal Omega v1.2.4 [43]. Subsequently, a logo showing conserved and variable regions among these sequences was generated using the WebLogo online tool v3.7.12 [44].

## 3. Results

The feature table consisted of 749 proteins derived from Sanger DNA sequencing of several fungal species exhibiting distinct susceptibility profiles to azoles (Supplementary Table S1). The DNA sequences available on NCBI were utilized with the Entrez direct tool to retrieve predicted and annotated CYP51 and ERG11 protein sequences based on the GenBank accession numbers of DNA sequences. In the case of *A. fumigatus* whole-genome sequences, a prediction step was necessary to identify CYP51 homologs from the 282 proteins directly obtained from NCBI. This approach yielded 340 WT and 409 NWT protein sequences characterized by their physiochemical properties using dedicated descriptors. The final feature table comprised 8772 columns, each representing a descriptor’s property (i.e., amino acid composition and hydrophobicity), and served as the foundation for constructing the ML model.

### 3.1. Machine Learning Model Construction

**Figure 2:**
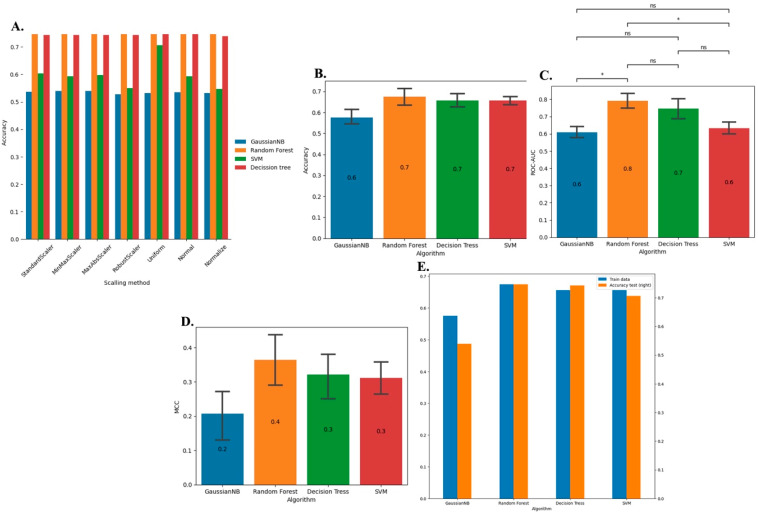
Modeling metrics. (**A**) Performance of scaling methods. (**B**) Accuracy of machine learning algorithms using Uniform, the best scaling method. (**C**) ROC–AUC of machine learning algorithms using Uniform, the best scaling method. *, *p*-value of ≤0.05; ns, *p*-value of >0.05 (Mann–Whitney multiple groups comparison with Bonferroni correction). (**D**) MCC of machine learning algorithms using Uniform, the best scaling method. (**E**) Comparison of accuracy metric between training and test datasets among machine learning algorithms.

The creation of the ML model involved choosing the optimal supervised algorithm and scaling method. Figure 2A illustrates the relationships between the scaling method and ML algorithms. Specifically, for GaussianNB, all the scalers exhibited nearly equal performance, with the MinMax scaler slightly outperforming the others. Both random forest and decision trees demonstrated similar effectiveness across all scalers, and the Standard scaler was selected for both algorithms. The Uniform scaler emerged as the most effective option for support vector machines (SVM) (Figure 2A–C).

After scaling the training datasets with the appropriate scalers for each ML algorithm, the performance of each algorithm was assessed using metrics such as accuracy, ROC–AUC, and MCC (Figure 2B). The accuracy was evaluated as the ratio of correct predictions to total predictions (CP/TP). The ROC–AUC score, which reflects the area under the receiver operating characteristic curve, provides insights into model specificity and sensitivity, with the following interpretations: 0.5–0.6 (failed), 0.6–0.7 (worthless), 0.7–0.8 (poor), 0.8–0.9 (good), and >0.9 (excellent) [45]. Finally, the MCC score which is mathematically expressed as MCC = (TP × TN − FP × FN)/(√((TP + FP) × (TP + FN) × (TN + FP) × (TN + FN)), in which TP = true positives, TN = true negatives, FN = false negatives, and FP = false positives, was considered. High MCC values are achieved when a model correctly predicts the majority of the metrics in a confusion matrix [45].

Random forest showed more significant accuracy variation than did other algorithms, with decision trees and SVM exhibiting similar average accuracy values. The GaussianNB method demonstrated lower accuracy than the other methods (Figure 2B). Regarding ROC–AUC scores, the random forest model achieved the highest score at 80%, followed by the decision trees at 70%. The GaussianNB and SVM models exhibited comparable average ROC–AUC scores at 60% (Figure 2C). Despite the lower MCC scores for all algorithms, the random forest algorithm outperformed the others, with a score of 40%, surpassing the decision tree (30%), SVM (30%), and GaussianNB (20%) algorithms (Figure 2D). Notably, compared with the algorithms, random forests exhibited balanced accuracy for training and test datasets compared to the other algorithms, indicating a greater probability of generalized predictions (Figure 2E).

However, the statistical analysis, which was conducted through Mann–Whitney multiple groups comparison with Bonferroni correction, did not reveal any significant differences at a *p*-value of ≤0.05 among the algorithms concerning accuracy, ROC–AUC, or MCC. This lack of significance is evident from the deviations observed in the bar plots (Figure 2B). Consequently, any of these algorithms can be employed for model training, expecting similar results. However, we decided to use the random forest due to its slight superiority in terms of accuracy variation, higher ROC–AUC, better MCC scores, and consistent accuracy across both the training and test datasets (Figure 2B–E). Concerning model optimization, the random forest model underwent training based on five cross-validation rounds, resulting in the identification of the best parameters: “bootstrap”: false, “criterion”: entropy, “max_depth”: 2, ”max_features”: log2, “min_samples_split”: 2, achieving a max accuracy score of approximately 72%.

### 3.2. Model Evaluation

**Figure 3:**
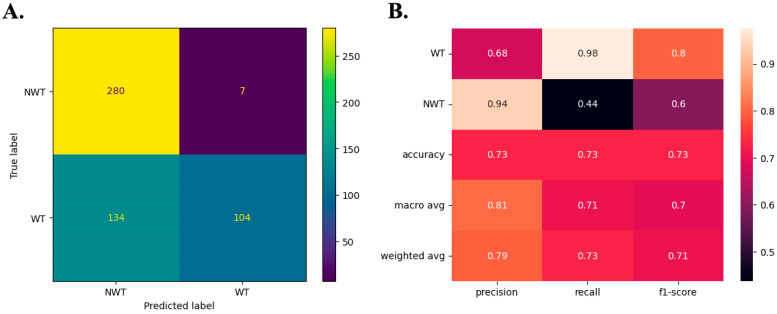
Metrics of supervised learning. (**A**) The confusion matrix shows correct and incorrect predictions for WT and NWT phenotypes, and (**B**) heatmap resuming the main classification metrics.

A confusion matrix is usually used to visualize the correct classification of labels. This study’s labels refer to the WT and NWT phenotypes of CYP51 and ERG11 protein sequences associated with azole resistance. The confusion matrix is presented in Figure 3A. As illustrated, among the 287 NWT protein sequences, the model incorrectly assigned seven sequences as having the WT phenotype. Conversely, out of 238 WT sequences, 134 were wrongly assigned as NWT phenotypes. These findings suggest that the model is more inclined to correctly identify NWT phenotypes than WT phenotypes (Figure 3A).

Figure 3B shows the classification results, illustrating key metrics for evaluating ML: accuracy, precision, recall, and f1-score. Precision, used to measure the proportion of truly positive predictions among those predicted as positive (defined as TP/(TP + FP), where TP and FP denote true positives and false positives, respectively), indicates that the model accurately predicted 94% of the NWT phenotypes and only 68% of the WT phenotypes (Figure 3B).

Recall, defined as TP/(TP + FN), where FN represents false negatives, aims to disclose the proportion of true positives not correctly predicted via the model. As depicted in Figure 3B, NWT’s (44%) recall is lower than WT’s (98%) recall. This suggests that, while the prediction of NWT sequences was highly accurate, the model may have overlooked many NWT sequences. These scores indicate that the model can correctly identify NWT sequences in a superior way than it can identify WT sequences. However, the model may have failed to identify some NWT sequences, contributing to an increased number of false-WT (false-negative) phenotypes. The f1-score, calculated as the harmonic mean of precision and recall (2*(Precision*Recall)/(Precision + Recall)), serves to harmonize measurements of precision and recall by assigning equal weight to both metrics, offering a comprehensive view of predictions. As depicted in Figure 3B, the f1-score for WT phenotype prediction was high at 80%. This suggests that although the model predicts the true phenotype for WT sequences with moderate precision, it can inaccurately predict some NWT sequences as WT sequences, as corroborated by the data in Figure 3A (indicating a high number of false-negative or false-WT phenotypes).

In contrast, the low recall for NWT prediction implies that the model may occasionally fail to identify all true NWT phenotypes in the dataset despite exhibiting high precision. Although the model can accurately identify NWT phenotypes, it might misclassify sequences with ambiguous or low-specific signatures as WT. Consequently, the precision in identifying WT sequences diminishes as the recall rate increases. This signifies that the reduction in precision primarily stems from an increase in false positives, specifically, false-WT sequences. The mean accuracy was 73%, indicating that most predictions were correct, considering the labeled test dataset.

Macro and weighted-average metrics represent each measurement’s arithmetic and weighted means across both classes (WT and NWT), respectively. Each class is assigned a weight based on the number of predicted sequences in the weighted average. As shown in Figure 3B, both averages are close for all the assessed metrics. This suggests that, despite some shortcomings in predicting specific phenotypes, either due to a loss of precision or an increase in false negatives (for NWT phenotypes) or false positives (for WT phenotypes), the model consistently achieved an average accuracy above 70%. Therefore, it can be considered suitable for discovering patterns associated with azole resistance in CYP51 and ERG11 sequences. The model also applied to screening CYP51 and ERG11 sequences lacking associated MIC data for classification. Additionally, it can be used to compare novel protein sequences using in silico methods.

### 3.3. Descriptors for the Classification of CYP51 and ERG11 Sequences

**Figure 4:**
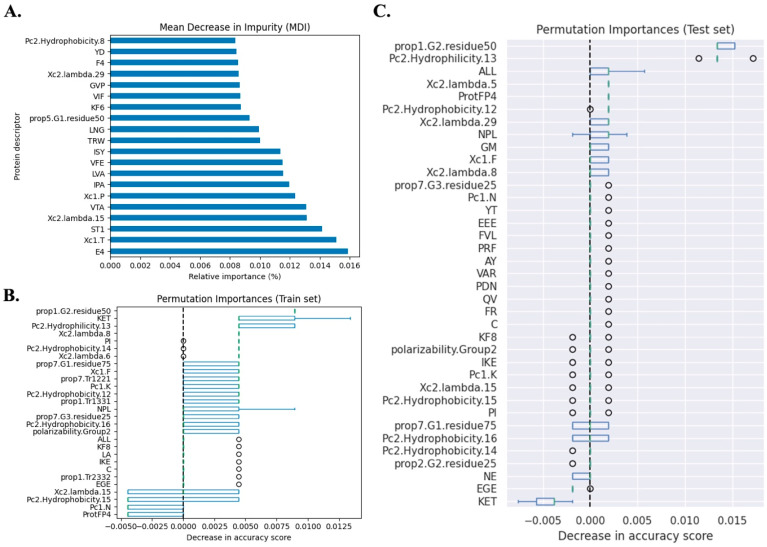
Most important (top 20) features’ attributes. (**A**) Main attributes related to WT and NWT phenotypes used in random forest classifier. Permutation importance analysis on (**B**) train and (**C**) test datasets.

The top 20 descriptors used for the classification of the CYP51 and ERG11 sequences are shown in Figure 4A. These descriptors, listed in descending order of relative importance, include physiochemical property descriptors based on multidimensional scaling (PCP) represented by the attribute E4 and pseudo-amino acid composition (PseAAC), with the attributes Xc1.T, Xc1.P, Xc2.lambda.15, and Xc2.lambda.29; structural topology scale (ST-scale) descriptor, represented by the attribute ST1; tripeptide composition descriptor, with the attributes VTA, IPA, LVA, VFE, ISY, TRW, LNG, VIF, and GVP; dipeptide composition descriptor, represented by the attribute YD; distribution descriptor, represented by prop.5.G1.residue.50; factor analysis descriptor, represented by the attribute F4; and amphiphilic pseudo-amino acid composition (APseAAC) descriptor, represented by the attribute Pc2.hydrophobicity.8.

Figure 4B illustrates the importance of the features in the training dataset, revealing variations in the tripeptide composition descriptors (KET, NPL, ALL, IKE, and EGE), distribution descriptors (prop1.G2.residue.50, prop7.G1.residue75, and prop.7.G3.residue25), APseAAC descriptors (Pc2.hydrophobicity.13, Pc2.hydrophobicity.14, Pc2.hydrophobicity.12, Pc2.hydrophobicity.16, Pc2.hydrophobicity.15, Pc1.K, and Pc1.N), PseAAC descriptors (Xc2.lambda.8, Xc2.lambda.6, Xc2.lambda.15, and Xc1.F), dipeptide descriptors (PI and LA), transition descriptors (prop7.Tr1221, prop1.Tr1331, and prop1.Tr2332), composition descriptor (polarizability.Group2), frequency of amino acid descriptor (attribute: C), Kidera factor descriptor (attribute: KF8), and ProtFP descriptor (attribute: ProtFP4).

Figure 4C presents the impact of the permutation shuffling of values in the test dataset. The main descriptors that varied included the distribution descriptor (attributes: prop1.G2.residue.50 and prop7.G1.residue.75), tripeptide frequency descriptor (attributes: ALL, NPL, and KET), PseAAC descriptor (attributes: Xc2.lambda.29, Xc1.F, and Xc2.lambda.8), dipeptide frequency descriptor (attributes: GM and NE), and APseAAC descriptor (attributes: Pc2.hydrophobicity.13 and Pc2.hydrophobicity.16).

Collectively, these descriptors and their attributes aim to address crucial questions: Does a chemical signature exist in these sequences that allows their characterization in terms of resistance? How might variations in their values impact the estimation? The first assignment was answered by elucidating the top 20 descriptor attributes in the dataset. The second assignment was approached through permutation analysis, where the values of all descriptor attributes were randomly varied, and the resultant effect on model accuracy was determined on both the training and test datasets (Figure 4B,C). Thus, combining the consensual importance of descriptor attributes, as inferred from the permutation analysis on both the training and test datasets, it becomes apparent that the variation induced by shuffling values in descriptors such as PseAAC, tripeptide composition, dipeptide composition, distribution descriptor, and APseAAC has the potential to describe hidden physiochemical properties in azole-resistant CYP51 and ERG11 proteins. These properties may explain minor differences between these sequences and shed light on their impact on fungal susceptibility to azoles.

### 3.4. Conservation among Sequences

A global alignment was conducted on all 749 selected protein sequences to address bias toward identifying NWT sequences. This analysis aimed to unveil the reasons behind the model’s evaluation metrics favoring NWT over WT. Then, a logo was created to visualize conserved motifs among the sequences and understand how such conservation might complicate the differentiation of closely related sequences associated with strains exhibiting different susceptibility levels to azole antifungals. Therefore, a biological interpretation of the model accuracy was facilitated by comparing global alignments, shedding light on the model’s robust discriminatory power in classifying NWT phenotypes compared to its ability to discern WT sequences.

**Figure 5:**
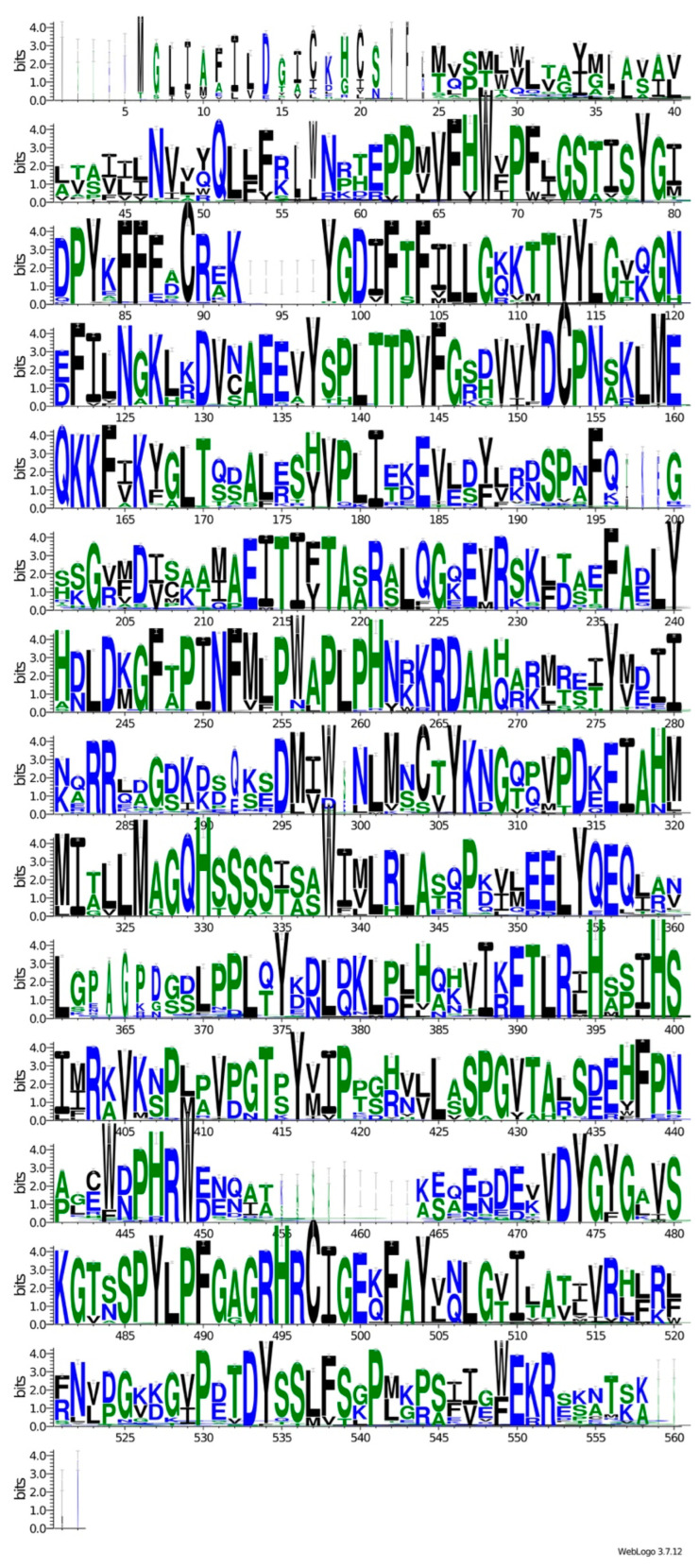
The protein logo shows the conservation degree of motifs shared by CYP51 and ERG11 amino acids’ sequences.

Figure 5 shows the conservation of amino acid positions in CYP51 and ERG11. The first five amino acids varied among the sequences, showing no discernible pattern. Similar variability is observed at positions 93–97, 455–463, and 559–562. Conversely, the remaining positions exhibit high conservation, with minimal variations in amino acid prevalence (e.g., position 40, position 186, and position 345) and complete conservation in specific sequences (e.g., position range of 160–164). The high degree of conservation among the sequences and sparse differences in amino acid composition at specific positions may explain the model’s inability to predict WT sequences, as protein sequences tend to be highly conserved (Figure 5).

## 4. Discussion

This study aimed to develop a machine learning (ML) model for identifying azole-resistant strains. The ML model discerns key features distinguishing between the susceptible (WT) and resistant (NWT) fungal strains, focusing on protein descriptors to gain insights into the molecular resistance mechanisms associated with CYP51/ERG11 protein sequences. Identifying these crucial features contributes to a deeper understanding of the biological factors influencing azole resistance, providing valuable insights for the scientific community.

The modeling process effectively mirrors real-world scenarios faced by researchers. The random forest algorithm was chosen for modeling because it demonstrated superior performance to GaussNB, decision trees, and support vector machines. Although there were no significant differences in accuracy among these algorithms, random forest demonstrated consistently superior results across multiple metrics used for model evaluation, including MCC and ROC–AUC analysis. This underscores its efficacy as the preferred option for predicting azole-resistant proteins.

In general, the optimized model presented satisfactory accuracy, aligning well with the challenges faced by wet-lab peers. This alignment is particularly crucial when dealing with conserved regions overlapping in CYP51/ERG11 protein sequences, as it may impair the differentiation of crucial attributes between the WT and NWT phenotypes. The achieved accuracy of 72% is deemed realistic, acknowledging that no model can correctly predict 100% of all the data. Conversely, an accuracy lower than 60% when the model is evaluated using testing data may reflect overfitting, a phenomenon caused by excessive learning from the training data, limiting its ability to generalize effectively to external datasets. Therefore, establishing literature guidelines on the minimal accuracy score for ML models is crucial because different fields may require distinct cutoff values. Analysts must exercise judgment in determining a suitable threshold for the model’s accuracy, considering the context of data acquisition, balancing, and structure [46]. It is also imperative to consider alternative metrics, such as the precision, recall, and f1-score, as they contribute to determining the effectiveness of the proposed model.

In this study, the ML model better predicted NWT sequences than WT sequences, primarily due to the high precision scores exhibited in NWT predictions. This observation was supported with the confusion matrix analysis, revealing a superior discriminatory ability in classifying NWT sequences. However, the recall for NWT predictions was lower than that for WT predictions, indicating that the model was somewhat restrictive in identifying specific NWT sequences incorrectly assigned as WT sequences. This increases the number of false-positive WT sequences, contributing to a decrease in precision for WT sequence predictions. Nevertheless, when comparing both the recall and precision scores summarized with the f1-score, the model demonstrated a tendency to discriminate between the WT and NWT phenotypes effectively in most instances. Furthermore, these test data may explain the comparatively lower performance of the model as determined via the MCC score on the training data (MCC score of 40%). The decrease in precision (true positives) is linked to this decrease in the MCC score despite the model presenting satisfactory (good) ROC–AUC and accuracy scores on the training datasets.

Despite using varied numerical values to describe the 749 proteins and constructing a substantial feature matrix with 8772 columns (attributes), discerning patterns proved challenging due to the high degree of conservation among the sequences. Nevertheless, even with the slight variation in amino acid composition, as illustrated by the protein logos, the model effectively classified many WT and NWT proteins. The metric scores used for model evaluation are deemed realistic.

It is important to acknowledge that the CYP51 and ERG11 sequences used in the present study originated from different fungal species, each of which presented distinct profiles of azole susceptibility. This implies that a particular isolate or strain may be resistant to voriconazole but susceptible to itraconazole and posaconazole or vice versa (Supplementary Table S1). Consequently, as several protein sequences presented different levels of susceptibility to different azole classes, this may account for the greater number of false-positive WT phenotypes, resulting in low precision for WT classification. In essence, the overlap of highly conserved sequences associated with different azole resistance profiles is a confounding factor. Additionally, the determination of MIC is subjective and visually assessed [4], introducing potential bias into the analysis. In certain instances, the MIC may be fungistatic rather than fungicidal [47]. Furthermore, the dataset comprised various publications with different references, such as CLSI and EUCAST, with the ECV values as the basis for the compilation. This approach might have led to inaccuracies in the dataset concerning NWT or WT strain classification. Despite diligent re-evaluation by comparing MICs to the reference guideline threshold, the analysis may have been influenced by the analyst’s subjectivity, potentially introducing inherent bias.

Fungal resistance is a complex process involving multiple factors [48] and cannot be underestimated. More than simply attributing a resistant or non-resistant phenotype to an entire microorganism based solely on the presence of a unique CYP51/ERG11 protein may be needed to understand the intricate mechanisms of resistance. Although distinctive substitution patterns in the amino acids of CYP51/ERG11 proteins are positively correlated with azole resistance [6], correlation alone does not necessarily indicate causality. Instead, it indicates the direction and strength of association, influenced by several factors [49], such as multifactorial resistance.

In the context of antifungal resistance, specific amino acid substitutions associated with resistant phenotypes may reflect a selection process favoring isoforms capable of adopting distinct conformational folds. This adaptation allows them to synergize with other mechanisms or metabolic pathways, effectively overcoming the stress induced by azole interference. Moving beyond this perspective, a feature importance analysis was used to determine whether shuffling a particular attribute leads to a decrease in an ML model’s accuracy (or another scoring metric). This analysis played a crucial role in identifying features offering insights into biological patterns related to azole resistance to various fungal proteins. This study evaluated the feature importance of both training and test data. In the initial scenario, the aim was to visualize the most crucial features for model learning. Conversely, in the second scenario, the emphasis shifted to visualizing the most significant features for model prediction. Combining the most crucial features from both scenarios made it feasible to estimate the optimal features capable of distinguishing between WT and NWT sequences.

As observed, the PseAAC, tripeptide composition, dipeptide composition, distribution descriptor, factor analysis descriptor, and APseAAC descriptor emerged as the most important features impacting model predictions. PseAAC is a method that considers the composition of the 20 amino acids in a protein sequence and incorporates the order in which they appear, utilizing a combination of discrete sequence correlation factors and the traditional amino acid composition [50]. Similarly, APseAAC combines sequence correlation factors to distinguish hydrophobic and hydrophilic distribution patterns in a protein sequence [50].

Composition descriptors, here summarized by the composition of amino acids (n = 20), dipeptides (n = 400), and tripeptides (n = 8000), calculated as the percentage of a given amino acid or combinations of amino acids (di- and tripeptides) concerning the entire sequence, serve as valuable parameters for condensing the information into a single value. This allows for comparing sequences with varying lengths and facilitates pattern extraction for ML analysis [51]. Furthermore, distribution descriptors are used to compute the percentage of neutral, polar, and hydrophobic residues along the length of a protein sequence [52].

Some studies investigating the 3D structure of the CYP51/ERG11 protein have revealed differences in the conformations of the WT and NWT proteins. Specifically, these variations involve the interaction mode of certain azoles’ lateral long-chains with 14-α-demethylase, affecting the channel that communicates substrates to the active sites of the protein. These active sites function as the loci for the interaction between azoles and the enzyme [10,53,54,55]. Consequently, studies on CYP51/ERG11 contribute significantly to understanding the mechanisms of azole resistance in fungi, reflecting a synergistic multifactorial process. Within this context, differences in the order and frequency of specific amino acids, along with their combinations in the sequences of CYP51/ERG11 WT and NWT phenotypes, may lead to tertiary conformation changes associated with patterns of hydrophobicity and hydrophilic regions. These alterations modify the active site of CYP51/ERG11. Docking analysis has revealed that CYP51 active site consists of hydrophobic amino acid residues that interact with the hydrophobic lanosterol (the precursor of ergosterol) through π-π and π-alkylation contacts with the amino acid residues [56]. Additionally, maintaining a balance between hydrophobic and hydrophilic characteristics is necessary to ensure that the substrate and active site interact in the CYP51/ERG11 channel. As demonstrated by previous studies, an increase in hydrophilicity reduces the affinity of these interactions [57,58].

## 5. Conclusions

Recognizing the limitations of traditional approaches, ML has emerged as a powerful tool for extracting valuable insights from complex and intricate biological datasets. The mathematical modeling of CYP51/ERG11 sequences to construct feature tables is crucial for revealing hidden physiochemical patterns within these sequences. In this study, we focused on protein attributes related to amino acid composition and their combination and hydrophobicity and hydrophilicity. This analysis revealed slight differences between NWT and WT proteins, highlighting significant molecular signatures. These findings have promising implications for future drug development strategies or in silico screening of potential NWT and WT lineages through comprehensive whole-genome analysis.

## Data Availability

The codes used in this study are available at https://github.com/Otavio20/CYPER accessed on 1 October 2023 along with the raw feature table of computed proteins’ descriptors.

## Supplementary Materials

The following supporting information can be downloaded at: https://www.mdpi.com/article/10.3390/microorganisms12081525/s1, Table S1: Metadata of datasets and respective publications selected in this study.
