# Supplementary material for: Harnessing Machine Learning to Uncover Hidden Patterns in Azole-Resistant CYP51/ERG11 Proteins
Source: Microorganisms. 2024 Jul 25;12(8):1525. doi: 10.3390/microorganisms12081525 (PMC11356363; doi:10.3390/microorganisms12081525)
Supplement: Supplementary file 1 [file microorganisms-12-01525-s001.zip › microorganisms-3124735-supplementary.pdf]

Supplementary Table S1. Metadata of datasets and respective publications selected in this study.

Phenotype	Especie	Type	*cyp* 51s	GenBank Acc. Ns	ProteinID	Publication
NWT	*A. flavus*	Filamentous	*cyp51A*	MT849811	QXL90911.1	https://doi.org/10.3390/genes11101217
NWT	*A. flavus*	Filamentous	*cyp51A*	MT849810	QXL90910.1	https://doi.org/10.3390/genes11101217
WT	*A. flavus*	Filamentous	*cyp51A*	MT849809	QXL90909.1	https://doi.org/10.3390/genes11101217
WT	*A. flavus*	Filamentous	*cyp51A*	MT849808	QXL90908.1	https://doi.org/10.3390/genes11101217
NWT	*A. flavus*	Filamentous	*cyp51A*	MT849807	QXL90907.1	https://doi.org/10.3390/genes11101217
WT	*A. flavus*	Filamentous	*cyp51A*	MT849806	QXL90906.1	https://doi.org/10.3390/genes11101217
WT	*A. flavus*	Filamentous	*cyp51A*	MT849805	QXL90905.1	https://doi.org/10.3390/genes11101217
WT	*A. flavus*	Filamentous	*cyp51A*	MT849804	QXL90904.1	https://doi.org/10.3390/genes11101217
WT	*A. flavus*	Filamentous	*cyp51A*	MT849803	QXL90903.1	https://doi.org/10.3390/genes11101217
WT	*A. flavus*	Filamentous	*cyp51A*	MT849802	QXL90902.1	https://doi.org/10.3390/genes11101217
NWT	*A. flavus*	Filamentous	*cyp51A*	MT849801	QXL90901.1	https://doi.org/10.3390/genes11101217
WT	*A. flavus*	Filamentous	*cyp51A*	MT849800	QXL90900.1	https://doi.org/10.3390/genes11101217
WT	*A. flavus*	Filamentous	*cyp51A*	MT849799	QXL90899.1	https://doi.org/10.3390/genes11101217
NWT	*A. flavus*	Filamentous	*cyp51A*	MT849798	QXL90898.1	https://doi.org/10.3390/genes11101217
WT	*A. flavus*	Filamentous	*cyp51A*	MT849797	QXL90897.1	https://doi.org/10.3390/genes11101217
NWT	*A. flavus*	Filamentous	*cyp51A*	MT849796	QXL90896.1	https://doi.org/10.3390/genes11101217
WT	*A. flavus*	Filamentous	*cyp51A*	MT849795	QXL90895.1	https://doi.org/10.3390/genes11101217
NWT	*A. flavus*	Filamentous	*cyp51A*	MT849794	QXL90894.1	https://doi.org/10.3390/genes11101217
NWT	*A. flavus*	Filamentous	*cyp51A*	MT849793	QXL90893.1	https://doi.org/10.3390/genes11101217
NWT	*A. flavus*	Filamentous	*cyp51B*	MT849771	QXL90871.1	https://doi.org/10.3390/genes11101217
NWT	*A. flavus*	Filamentous	*cyp51B*	MT849772	QXL90872.1	https://doi.org/10.3390/genes11101217
WT	*A. flavus*	Filamentous	*cyp51B*	MT849773	QXL90873.1	https://doi.org/10.3390/genes11101217
WT	*A. flavus*	Filamentous	*cyp51B*	MT849774	QXL90874.1	https://doi.org/10.3390/genes11101217
NWT	*A. flavus*	Filamentous	*cyp51B*	MT849775	QXL90875.1	https://doi.org/10.3390/genes11101217
WT	*A. flavus*	Filamentous	*cyp51B*	MT849776	QXL90876.1	https://doi.org/10.3390/genes11101217
WT	*A. flavus*	Filamentous	*cyp51B*	MT849777	QXL90877.1	https://doi.org/10.3390/genes11101217
WT	*A. flavus*	Filamentous	*cyp51B*	MT849778	QXL90878.1	https://doi.org/10.3390/genes11101217
WT	*A. flavus*	Filamentous	*cyp51B*	MT849779	QXL90879.1	https://doi.org/10.3390/genes11101217
WT	*A. flavus*	Filamentous	*cyp51B*	MT849780	QXL90880.1	https://doi.org/10.3390/genes11101217
NWT	*A. flavus*	Filamentous	*cyp51B*	MT849781	QXL90881.1	https://doi.org/10.3390/genes11101217
WT	*A. flavus*	Filamentous	*cyp51B*	MT849782	QXL90882.1	https://doi.org/10.3390/genes11101217
WT	*A. flavus*	Filamentous	*cyp51B*	MT849783	QXL90883.1	https://doi.org/10.3390/genes11101217
NWT	*A. flavus*	Filamentous	*cyp51B*	MT849784	QXL90884.1	https://doi.org/10.3390/genes11101217
WT	*A. flavus*	Filamentous	*cyp51B*	MT849785	QXL90885.1	https://doi.org/10.3390/genes11101217
NWT	*A. flavus*	Filamentous	*cyp51B*	MT849786	QXL90886.1	https://doi.org/10.3390/genes11101217
WT	*A. flavus*	Filamentous	*cyp51B*	MT849787	QXL90887.1	https://doi.org/10.3390/genes11101217
NWT	*A. flavus*	Filamentous	*cyp51B*	MT849789	QXL90889.1	https://doi.org/10.3390/genes11101217
WT	*A. flavus*	Filamentous	*cyp51B*	MT849791	QXL90891.1	https://doi.org/10.3390/genes11101217
NWT	*A. flavus*	Filamentous	*cyp51C*	MT853225	QXL90913.1	https://doi.org/10.3390/genes11101217
NWT	*A. flavus*	Filamentous	*cyp51C*	MT853226	QXL90914.1	https://doi.org/10.3390/genes11101217
WT	*A. flavus*	Filamentous	*cyp51C*	MT853227	QXL90915.1	https://doi.org/10.3390/genes11101217
WT	*A. flavus*	Filamentous	*cyp51C*	MT853228	QXL90916.1	https://doi.org/10.3390/genes11101217

NWT	*A. flavus*	Filamentous	*cyp51C*	MT853229	QXL90917.1	https://doi.org/10.3390/genes11101217
WT	*A. flavus*	Filamentous	*cyp51C*	MT853230	QXL90918.1	https://doi.org/10.3390/genes11101217
WT	*A. flavus*	Filamentous	*cyp51C*	MT853231	QXL90919.1	https://doi.org/10.3390/genes11101217
WT	*A. flavus*	Filamentous	*cyp51C*	MT853233	QXL90920.1	https://doi.org/10.3390/genes11101217
WT	*A. flavus*	Filamentous	*cyp51C*	MT853234	QXL90921.1	https://doi.org/10.3390/genes11101217
NWT	*A. flavus*	Filamentous	*cyp51C*	MT853235	QXL90922.1	https://doi.org/10.3390/genes11101217
WT	*A. flavus*	Filamentous	*cyp51C*	MT853236	QXL90923.1	https://doi.org/10.3390/genes11101217
WT	*A. flavus*	Filamentous	*cyp51C*	MT853237	QXL90924.1	https://doi.org/10.3390/genes11101217
NWT	*A. flavus*	Filamentous	*cyp51C*	MT853238	QXL90925.1	https://doi.org/10.3390/genes11101217
WT	*A. flavus*	Filamentous	*cyp51C*	MT853239	QXL90926.1	https://doi.org/10.3390/genes11101217
NWT	*A. flavus*	Filamentous	*cyp51C*	MT853240	QXL90927.1	https://doi.org/10.3390/genes11101217
WT	*A. flavus*	Filamentous	*cyp51C*	MT853241	QXL90928.1	https://doi.org/10.3390/genes11101217
NWT	*A. flavus*	Filamentous	*cyp51C*	MT853242	QXL90929.1	https://doi.org/10.3390/genes11101217
NWT	*A. flavus*	Filamentous	*cyp51C*	MT853243	QXL90930.1	https://doi.org/10.3390/genes11101217
NWT	*A. flavus*	Filamentous	*cyp51C*	MT853244	QXL90931.1	https://doi.org/10.3390/genes11101217
WT	*A. flavus*	Filamentous	*cyp51C*	MT853245	QXL90932.1	https://doi.org/10.3390/genes11101217
WT	*A. fumigatus*	Filamentous	cyp51A	MT418924	QJI54838.1	https://doi.org/10.1016/j.jhazmat.2020.123200
WT	*A. fumigatus*	Filamentous	cyp51A	MT418926	QJI54840.1	https://doi.org/10.1016/j.jhazmat.2020.123200
WT	*A. fumigatus*	Filamentous	cyp51A	MT418920	QJI54834.1	https://doi.org/10.1016/j.jhazmat.2020.123200
WT	*A. fumigatus*	Filamentous	cyp51A	MT418923	QJI54837.1	https://doi.org/10.1016/j.jhazmat.2020.123200
WT	*A. fumigatus*	Filamentous	cyp51A	MT418918	QJI54832.1	https://doi.org/10.1016/j.jhazmat.2020.123200
WT	*A. fumigatus*	Filamentous	cyp51A	MT418917	QJI54831.1	https://doi.org/10.1016/j.jhazmat.2020.123200
NWT	*A. fumigatus*	Filamentous	cyp51A	MT418921	QJI54835.1	https://doi.org/10.1016/j.jhazmat.2020.123200
NWT	*A. fumigatus*	Filamentous	cyp51A	MT418927	QJI54841.1	https://doi.org/10.1016/j.jhazmat.2020.123200
NWT	*A. fumigatus*	Filamentous	cyp51A	MT418925	QJI54839.1	https://doi.org/10.1016/j.jhazmat.2020.123200
NWT	*A. fumigatus*	Filamentous	cyp51A	MT418928	QJI54842.1	https://doi.org/10.1016/j.jhazmat.2020.123200
NWT	*A. fumigatus*	Filamentous	cyp51A	MT418922	QJI54836.1	https://doi.org/10.1016/j.jhazmat.2020.123200
NWT	*A. fumigatus*	Filamentous	cyp51A	MT418919	QJI54833.1	https://doi.org/10.1016/j.jhazmat.2020.123200
WT	*A. tubingensis*	Filamentous	cyp51A	QCL08193.1	QCL08193.1	https://doi.org/10.1128/jcm.02023-18
WT	*A. tubingensis*	Filamentous	cyp51A	QCL08194.1	QCL08194.1	https://doi.org/10.1128/jcm.02023-18
WT	*A. tubingensis*	Filamentous	cyp51A	QCL08195.1	QCL08195.1	https://doi.org/10.1128/jcm.02023-18
WT	*A. tubingensis*	Filamentous	cyp51A	QCL08196.1	QCL08196.1	https://doi.org/10.1128/jcm.02023-18
WT	*A. tubingensis*	Filamentous	cyp51A	QCL08197.1	QCL08197.1	https://doi.org/10.1128/jcm.02023-18
WT	*A. tubingensis*	Filamentous	cyp51A	QCL08198.1	QCL08198.1	https://doi.org/10.1128/jcm.02023-18
WT	*A. tubingensis*	Filamentous	cyp51A	QCL08199.1	QCL08199.1	https://doi.org/10.1128/jcm.02023-18
WT	*A. tubingensis*	Filamentous	cyp51A	QCL08200.1	QCL08200.1	https://doi.org/10.1128/jcm.02023-18
WT	*A. tubingensis*	Filamentous	cyp51A	QCL08201.1	QCL08201.1	https://doi.org/10.1128/jcm.02023-18
WT	*A. tubingensis*	Filamentous	cyp51A	QCL08202.1	QCL08202.1	https://doi.org/10.1128/jcm.02023-18
WT	*A. tubingensis*	Filamentous	cyp51A	QCL08203.1	QCL08203.1	https://doi.org/10.1128/jcm.02023-18
WT	*A. tubingensis*	Filamentous	cyp51A	QCL08204.1	QCL08204.1	https://doi.org/10.1128/jcm.02023-18
WT	*A. niger*	Filamentous	cyp51A	QCL08205.1	QCL08205.1	https://doi.org/10.1128/jcm.02023-18
NWT	*A. niger*	Filamentous	cyp51A	QCL08206.1	QCL08206.1	https://doi.org/10.1128/jcm.02023-18
WT	*A. niger*	Filamentous	cyp51A	QCL08207.1	QCL08207.1	https://doi.org/10.1128/jcm.02023-18
WT	*A. niger*	Filamentous	cyp51A	QCL08208.1	QCL08208.1	https://doi.org/10.1128/jcm.02023-18
WT	*A. niger*	Filamentous	cyp51A	QCL08209.1	QCL08209.1	https://doi.org/10.1128/jcm.02023-18
WT	*A. niger*	Filamentous	cyp51A	QCL08210.1	QCL08210.1	https://doi.org/10.1128/jcm.02023-18
WT	*A. niger*	Filamentous	cyp51A	QCL08211.1	QCL08211.1	https://doi.org/10.1128/jcm.02023-18
WT	*A. niger*	Filamentous	cyp51A	QCL08212.1	QCL08212.1	https://doi.org/10.1128/jcm.02023-18

WT	*A. niger*	Filamentous	cyp51A	QCL08213.1	QCL08213.1	https://doi.org/10.1128/jcm.02023-18
WT	*A. niger*	Filamentous	cyp51A	QCL08214.1	QCL08214.1	https://doi.org/10.1128/jcm.02023-18
WT	*A. niger*	Filamentous	cyp51A	QCL08215.1	QCL08215.1	https://doi.org/10.1128/jcm.02023-18
WT	*A. fumigatus*	Filamentous	cyp51A	QCL08162.1	QCL08162.1	https://doi.org/10.1128/jcm.02023-18
NWT	*A. fumigatus*	Filamentous	cyp51A	QCL08163.1	QCL08163.1	https://doi.org/10.1128/jcm.02023-18
WT	*A. fumigatus*	Filamentous	cyp51A	QCL08164.1	QCL08164.1	https://doi.org/10.1128/jcm.02023-18
WT	*A. fumigatus*	Filamentous	cyp51A	QCL08165.1	QCL08165.1	https://doi.org/10.1128/jcm.02023-18
WT	*A. fumigatus*	Filamentous	cyp51A	QCL08166.1	QCL08166.1	https://doi.org/10.1128/jcm.02023-18
WT	*A. fumigatus*	Filamentous	cyp51A	QCL08167.1	QCL08167.1	https://doi.org/10.1128/jcm.02023-18
WT	*A. fumigatus*	Filamentous	cyp51A	QCL08168.1	QCL08168.1	https://doi.org/10.1128/jcm.02023-18
WT	*A. fumigatus*	Filamentous	cyp51A	QCL08169.1	QCL08169.1	https://doi.org/10.1128/jcm.02023-18
WT	*A. fumigatus*	Filamentous	cyp51A	QCL08170.1	QCL08170.1	https://doi.org/10.1128/jcm.02023-18
WT	*A. fumigatus*	Filamentous	cyp51A	QCL08171.1	QCL08171.1	https://doi.org/10.1128/jcm.02023-18
WT	*A. fumigatus*	Filamentous	cyp51A	QCL08172.1	QCL08172.1	https://doi.org/10.1128/jcm.02023-18
WT	*A. fumigatus*	Filamentous	cyp51A	QCL08173.1	QCL08173.1	https://doi.org/10.1128/jcm.02023-18
WT	*A. fumigatus*	Filamentous	cyp51A	QCL08174.1	QCL08174.1	https://doi.org/10.1128/jcm.02023-18
WT	*A. fumigatus*	Filamentous	cyp51A	QCL08175.1	QCL08175.1	https://doi.org/10.1128/jcm.02023-18
WT	*A. fumigatus*	Filamentous	cyp51A	QCL08176.1	QCL08176.1	https://doi.org/10.1128/jcm.02023-18
WT	*A. fumigatus*	Filamentous	cyp51A	QCL08177.1	QCL08177.1	https://doi.org/10.1128/jcm.02023-18
WT	*A. fumigatus*	Filamentous	cyp51A	QCL08178.1	QCL08178.1	https://doi.org/10.1128/jcm.02023-18
WT	*A. fumigatus*	Filamentous	cyp51A	QCL08179.1	QCL08179.1	https://doi.org/10.1128/jcm.02023-18
WT	*A. fumigatus*	Filamentous	cyp51A	QCL08180.1	QCL08180.1	https://doi.org/10.1128/jcm.02023-18
WT	*A. fumigatus*	Filamentous	cyp51A	QCL08181.1	QCL08181.1	https://doi.org/10.1128/jcm.02023-18
WT	*A. awamori*	Filamentous	cyp51A	QCL08182.1	QCL08182.1	https://doi.org/10.1128/jcm.02023-18
WT	*A. awamori*	Filamentous	cyp51A	QCL08183.1	QCL08183.1	https://doi.org/10.1128/jcm.02023-18
WT	*A. awamori*	Filamentous	cyp51A	QCL08184.1	QCL08184.1	https://doi.org/10.1128/jcm.02023-18
WT	*A. awamori*	Filamentous	cyp51A	QCL08185.1	QCL08185.1	https://doi.org/10.1128/jcm.02023-18
WT	*A. awamori*	Filamentous	cyp51A	QCL08186.1	QCL08186.1	https://doi.org/10.1128/jcm.02023-18
WT	*A. awamori*	Filamentous	cyp51A	QCL08187.1	QCL08187.1	https://doi.org/10.1128/jcm.02023-18
WT	*A. awamori*	Filamentous	cyp51A	QCL08188.1	QCL08188.1	https://doi.org/10.1128/jcm.02023-18
WT	*A. awamori*	Filamentous	cyp51A	QCL08189.1	QCL08189.1	https://doi.org/10.1128/jcm.02023-18
WT	*A. awamori*	Filamentous	cyp51A	QCL08190.1	QCL08190.1	https://doi.org/10.1128/jcm.02023-18
WT	*A. awamori*	Filamentous	cyp51A	QCL08191.1	QCL08191.1	https://doi.org/10.1128/jcm.02023-18
WT	*A. awamori*	Filamentous	cyp51A	QCL08192.1	QCL08192.1	https://doi.org/10.1128/jcm.02023-18
WT	*A. lucuhensis*	Filamentous	cyp51A	QCL08216.1	QCL08216.1	https://doi.org/10.1128/jcm.02023-18
WT	*A. lucuhensis*	Filamentous	cyp51A	QCL08217.1	QCL08217.1	https://doi.org/10.1128/jcm.02023-18
NWT	*A. fumigatus*	Filamentous	cyp51A	AAF32372.1	AAF32372.1	https://doi.org/10.1080/mmy.39.3.299.302
NWT	*C. parapsilopsis*	Yeast	ERG11	MW714301	QZA57185.1	https://doi.org/10.3390%2Fjof7040259
NWT	*C. parapsilopsis*	Yeast	ERG11	MW714302	QZA57186.1	https://doi.org/10.3390%2Fjof7040259
NWT	*C. parapsilopsis*	Yeast	ERG11	MW714303	QZA57187.1	https://doi.org/10.3390%2Fjof7040259
NWT	*C. parapsilopsis*	Yeast	ERG11	MW714304	QZA57188.1	https://doi.org/10.3390%2Fjof7040259
NWT	*C. parapsilopsis*	Yeast	ERG11	MW714305	QZA57189.1	https://doi.org/10.3390%2Fjof7040259
NWT	*C. parapsilopsis*	Yeast	ERG11	MW714306	QZA57190.1	https://doi.org/10.3390%2Fjof7040259
NWT	*C. parapsilopsis*	Yeast	ERG11	MW714307	QZA57191.1	https://doi.org/10.3390%2Fjof7040259
NWT	*C. parapsilopsis*	Yeast	ERG11	MW714308	QZA57192.1	https://doi.org/10.3390%2Fjof7040259
WT	*A. fumigatus*	Filamentous	CYP51A	XP_749134.1	XP_749134.1	https://www.ncbi.nlm.nih.gov/pmc/articles/PMC670945 2/
NWT	*A. fumigatus*	Filamentous	CYP51A	QOV09152.1	QOV09152.1	https://doi.org/10.3389/fmicb.2020.599233
WT	*C. glabrata*	Yeast	ERG11	KR998002	AMR44138.1	https://doi.org/10.1590%2F0074-02760150400

NWT	*C. glabrata*	Yeast	ERG11	KR998003	AMR44139.1	https://doi.org/10.1590%2F0074-02760150400
NWT	*C. glabrata*	Yeast	ERG11	KR998004	AMR44140.1	https://doi.org/10.1590%2F0074-02760150400
NWT	*C. glabrata*	Yeast	ERG11	KR998005	AMR44141.1	https://doi.org/10.1590%2F0074-02760150400
NWT	*C. glabrata*	Yeast	ERG11	KR998006	AMR44142.1	https://doi.org/10.1590%2F0074-02760150400
NWT	*C. glabrata*	Yeast	ERG11	KR998007	AMR44143.1	https://doi.org/10.1590%2F0074-02760150400
NWT	*C. glabrata*	Yeast	ERG11	KR998008	AMR44144.1	https://doi.org/10.1590%2F0074-02760150400
NWT	*C. glabrata*	Yeast	ERG11	KR998009	AMR44145.1	https://doi.org/10.1590%2F0074-02760150400
NWT	*C. glabrata*	Yeast	ERG11	KR998010	AMR44146.1	https://doi.org/10.1590%2F0074-02760150400
WT	*C. krusei*	Yeast	ERG11	KR998011	AMR44147.1	https://doi.org/10.1590%2F0074-02760150400
NWT	*C. krusei*	Yeast	ERG11	KR998012	AMR44148.1	https://doi.org/10.1590%2F0074-02760150400
NWT	*C. krusei*	Yeast	ERG11	KR998013	AMR44149.1	https://doi.org/10.1590%2F0074-02760150400
NWT	*C. krusei*	Yeast	ERG11	KR998014	AMR44150.1	https://doi.org/10.1590%2F0074-02760150400
WT	*C. tropicalis*	Yeast	ERG11	KR998015	AMR44151.1	https://doi.org/10.1590%2F0074-02760150400
NWT	*C. tropicalis*	Yeast	ERG11	KR998016	AMR44152.1	https://doi.org/10.1590%2F0074-02760150400
NWT	*C. tropicalis*	Yeast	ERG11	KR998017	AMR44153.1	https://doi.org/10.1590%2F0074-02760150400
NWT	*C. tropicalis*	Yeast	ERG11	KR998018	AMR44154.1	https://doi.org/10.1590%2F0074-02760150400
WT	*C. glabrata*	Yeast	ERG11	DQ060157	AAY59417.1	https://doi.org/10.1128/AAC.01384-08
WT	*C. glabrata*	Yeast	ERG11	FJ167408	ACI24046.1	https://doi.org/10.1128/AAC.01384-08
NWT	*C. glabrata*	Yeast	ERG11	FJ167409	ACI24047.1	https://doi.org/10.1128/AAC.01384-08
NWT	*A. fumigatus*	Filamentous	CYP51A	MT108465	QKI35924.1	https://doi.org/10.1099/jmm.0.001200
NWT	*A. fumigatus*	Filamentous	CYP51A	MT108466	QKI35925.1	https://doi.org/10.1099/jmm.0.001200
NWT	*A. fumigatus*	Filamentous	CYP51A	MT108467	QKI35926.1	https://doi.org/10.1099/jmm.0.001200
NWT	*A. fumigatus*	Filamentous	CYP51A	MT108468	QKI35927.1	https://doi.org/10.1099/jmm.0.001200
NWT	*A. fumigatus*	Filamentous	CYP51A	MT108469	QKI35928.1	https://doi.org/10.1099/jmm.0.001200
NWT	*A. fumigatus*	Filamentous	CYP51A	MT108470	QKI35929.1	https://doi.org/10.1099/jmm.0.001200
WT	*A. fumigatus*	Filamentous	CYP51A	MT108471	QKI35930.1	https://doi.org/10.1099/jmm.0.001200
NWT	*A. fumigatus*	Filamentous	CYP51A	MT108472	QKI35931.1	https://doi.org/10.1099/jmm.0.001200
NWT	*A. fumigatus*	Filamentous	CYP51A	MT108473	QKI35932.1	https://doi.org/10.1099/jmm.0.001200
NWT	*A. fumigatus*	Filamentous	CYP51A	MT108474	QKI35933.1	https://doi.org/10.1099/jmm.0.001200
NWT	*A. fumigatus*	Filamentous	CYP51A	QHB15419.1	QHB15419.1	https://doi.org/10.1128/aac.02059-19
NWT	*A. fumigatus*	Filamentous	CYP51A	QHB15418.1	QHB15418.1	https://doi.org/10.1128/aac.02059-19
NWT	*A. fumigatus*	Filamentous	CYP51A	MK417817	QFO37883.1	https://doi.org/10.1111/jam.14217
NWT	*A. fumigatus*	Filamentous	CYP51A	MK417818	QFO37884.1	https://doi.org/10.1111/jam.14217
NWT	*A. fumigatus*	Filamentous	CYP51A	MK417819	QFO37885.1	https://doi.org/10.1111/jam.14217
NWT	*A. fumigatus*	Filamentous	CYP51A	MK417820	QFO37886.1	https://doi.org/10.1111/jam.14217
NWT	*A. fumigatus*	Filamentous	CYP51A	MK417821	QFO37887.1	https://doi.org/10.1111/jam.14217
NWT	*A. fumigatus*	Filamentous	CYP51A	MK417822	QFO37888.1	https://doi.org/10.1111/jam.14217
NWT	*A. fumigatus*	Filamentous	CYP51A	MK417823	QFO37889.1	https://doi.org/10.1111/jam.14217
NWT	*A. fumigatus*	Filamentous	CYP51A	MK417824	QFO37890.1	https://doi.org/10.1111/jam.14217
NWT	*A. fumigatus*	Filamentous	CYP51A	MK417825	QFO37891.1	https://doi.org/10.1111/jam.14217
NWT	*A. fumigatus*	Filamentous	CYP51A	MK417826	QFO37892.1	https://doi.org/10.1111/jam.14217
NWT	*A. fumigatus*	Filamentous	CYP51A	MK417827	QFO37893.1	https://doi.org/10.1111/jam.14217
NWT	*A. fumigatus*	Filamentous	CYP51A	MK417828	QFO37894.1	https://doi.org/10.1111/jam.14217
NWT	*A. fumigatus*	Filamentous	CYP51A	MH510860	QAW56499.1	https://doi.org/10.1016/j.jcf.2019.01.001
NWT	*A. fumigatus*	Filamentous	CYP51A	MH510861	QAW56500.1	https://doi.org/10.1016/j.jcf.2019.01.001
NWT	*A. fumigatus*	Filamentous	CYP51A	MH510862	QAW56501.1	https://doi.org/10.1016/j.jcf.2019.01.001
NWT	*A. fumigatus*	Filamentous	CYP51A	MH510863	QAW56502.1	https://doi.org/10.1016/j.jcf.2019.01.001
NWT	*A. fumigatus*	Filamentous	CYP51A	MH510864	QAW56503.1	https://doi.org/10.1016/j.jcf.2019.01.001
WT	*C. parapsilopsis*	Yeast	ERG11	GQ302972	ACT67904.1	https://doi.org/10.1128/AAC.01177-15

NWT	*C. parapsilopsis*	Yeast	ERG11	KR082784	AKU41523.1	https://doi.org/10.1128/AAC.01177-15
NWT	*C. parapsilopsis*	Yeast	ERG11	KR082785	AKU41524.1	https://doi.org/10.1128/AAC.01177-15
NWT	*C. parapsilopsis*	Yeast	ERG11	KR082786	AKU41525.1	https://doi.org/10.1128/AAC.01177-15
NWT	*C. parapsilopsis*	Yeast	ERG11	KR082787	AKU41526.1	https://doi.org/10.1128/AAC.01177-15
NWT	*C. parapsilopsis*	Yeast	ERG11	KR082788	AKU41527.1	https://doi.org/10.1128/AAC.01177-15
NWT	*C. parapsilopsis*	Yeast	ERG11	KR082789	AKU41528.1	https://doi.org/10.1128/AAC.01177-15
NWT	*C. parapsilopsis*	Yeast	ERG11	KR082790	AKU41529.1	https://doi.org/10.1128/AAC.01177-15
NWT	*C. parapsilopsis*	Yeast	ERG11	KR082791	AKU41530.1	https://doi.org/10.1128/AAC.01177-15
NWT	*C. parapsilopsis*	Yeast	ERG11	KR082792	AKU41531.1	https://doi.org/10.1128/AAC.01177-15
NWT	*A. flavus*	Filamentous	CYP51C	KR822399	AKQ20794.1	https://doi.org/10.1128/AAC.00637-15
WT	*A. flavus*	Filamentous	CYP51C	KR822400	AKQ20795.1	https://doi.org/10.1128/AAC.00637-15
WT	*C. tropicalis*	Yeast	ERG11	MW015968	QQY97041.1	https://doi.org/10.1371%2Fjournal.pone.0245160
WT	*C. tropicalis*	Yeast	ERG11	MW015969	QQY97042.1	https://doi.org/10.1371%2Fjournal.pone.0245160
WT	*C. tropicalis*	Yeast	ERG11	MW015970	QQY97043.1	https://doi.org/10.1371%2Fjournal.pone.0245160
WT	*C. tropicalis*	Yeast	ERG11	MW015971	QQY97044.1	https://doi.org/10.1371%2Fjournal.pone.0245160
WT	*C. tropicalis*	Yeast	ERG11	MW015972	QQY97045.1	https://doi.org/10.1371%2Fjournal.pone.0245160
WT	*C. tropicalis*	Yeast	ERG11	MW015973	QQY97046.1	https://doi.org/10.1371%2Fjournal.pone.0245160
NWT	*C. tropicalis*	Yeast	ERG11	MW015974	QQY97047.1	https://doi.org/10.1371%2Fjournal.pone.0245160
WT	*C. tropicalis*	Yeast	ERG11	MW015975	QQY97048.1	https://doi.org/10.1371%2Fjournal.pone.0245160
WT	*C. tropicalis*	Yeast	ERG11	MW015976	QQY97049.1	https://doi.org/10.1371%2Fjournal.pone.0245160
WT	*C. tropicalis*	Yeast	ERG11	MW015977	QQY97050.1	https://doi.org/10.1371%2Fjournal.pone.0245160
NWT	*C. tropicalis*	Yeast	ERG11	MW015978	QQY97051.1	https://doi.org/10.1371%2Fjournal.pone.0245160
WT	*C. tropicalis*	Yeast	ERG11	MW015979	QQY97052.1	https://doi.org/10.1371%2Fjournal.pone.0245160
WT	*C. tropicalis*	Yeast	ERG11	MW015980	QQY97053.1	https://doi.org/10.1371%2Fjournal.pone.0245160
WT	*C. tropicalis*	Yeast	ERG11	MW015981	QQY97054.1	https://doi.org/10.1371%2Fjournal.pone.0245160
NWT	*C. tropicalis*	Yeast	ERG11	MW015982	QQY97055.1	https://doi.org/10.1371%2Fjournal.pone.0245160
NWT	*C. tropicalis*	Yeast	ERG11	MW015983	QQY97056.1	https://doi.org/10.1371%2Fjournal.pone.0245160
NWT	*C. tropicalis*	Yeast	ERG11	MW015984	QQY97057.1	https://doi.org/10.1371%2Fjournal.pone.0245160
WT	*C. tropicalis*	Yeast	ERG11	MW015985	QQY97058.1	https://doi.org/10.1371%2Fjournal.pone.0245160
NWT	*C. tropicalis*	Yeast	ERG11	MW015986	QQY97059.1	https://doi.org/10.1371%2Fjournal.pone.0245160
NWT	*C. tropicalis*	Yeast	ERG11	MW015956	QQY97029.1	https://doi.org/10.1371%2Fjournal.pone.0245160
NWT	*C. tropicalis*	Yeast	ERG11	MW015957	QQY97030.1	https://doi.org/10.1371%2Fjournal.pone.0245160
NWT	*C. tropicalis*	Yeast	ERG11	MW015958	QQY97031.1	https://doi.org/10.1371%2Fjournal.pone.0245160
NWT	*C. tropicalis*	Yeast	ERG11	MW015959	QQY97032.1	https://doi.org/10.1371%2Fjournal.pone.0245160
NWT	*C. tropicalis*	Yeast	ERG11	MW015960	QQY97033.1	https://doi.org/10.1371%2Fjournal.pone.0245160
NWT	*C. tropicalis*	Yeast	ERG11	MW015961	QQY97034.1	https://doi.org/10.1371%2Fjournal.pone.0245160
NWT	*C. tropicalis*	Yeast	ERG11	MW015962	QQY97035.1	https://doi.org/10.1371%2Fjournal.pone.0245160
NWT	*C. tropicalis*	Yeast	ERG11	MW015963	QQY97036.1	https://doi.org/10.1371%2Fjournal.pone.0245160
NWT	*C. tropicalis*	Yeast	ERG11	MW015964	QQY97037.1	https://doi.org/10.1371%2Fjournal.pone.0245160
NWT	*C. tropicalis*	Yeast	ERG11	MW015965	QQY97038.1	https://doi.org/10.1371%2Fjournal.pone.0245160
NWT	*C. tropicalis*	Yeast	ERG11	MW015966	QQY97039.1	https://doi.org/10.1371%2Fjournal.pone.0245160
NWT	*C. tropicalis*	Yeast	ERG11	MW015967	QQY97040.1	https://doi.org/10.1371%2Fjournal.pone.0245160
WT	*C. tropicalis*	Yeast	ERG11	KP938783	ALA65470.1	https://doi.org/10.1016%2Fj.mmcr.2016.02.001
WT	*C. tropicalis*	Yeast	ERG11	KP938784	ALA65471.1	https://doi.org/10.1016%2Fj.mmcr.2016.02.001
WT	*C. tropicalis*	Yeast	ERG11	KP938785	ALA65472.1	https://doi.org/10.1016%2Fj.mmcr.2016.02.001
WT	*C. tropicalis*	Yeast	ERG11	KP938786	ALA65473.1	https://doi.org/10.1016%2Fj.mmcr.2016.02.001
NWT	*C. tropicalis*	Yeast	ERG11	KP938787	ALA65474.1	https://doi.org/10.1016%2Fj.mmcr.2016.02.001
NWT	*C. tropicalis*	Yeast	ERG11	KP938788	ALA65475.1	https://doi.org/10.1016%2Fj.mmcr.2016.02.001
NWT	*C. tropicalis*	Yeast	ERG11	KP938789	ALA65476.1	https://doi.org/10.1016%2Fj.mmcr.2016.02.001

NWT	*C. tropicalis*	Yeast	ERG11	KP938790	ALA65477.1	https://doi.org/10.1016%2Fj.mmcr.2016.02.001
WT	*C. tropicalis*	Yeast	ERG11	KC542323	AGT98513.1	https://doi.org/10.1128%2FAAC.00477-13
NWT	*C. tropicalis*	Yeast	ERG11	KC542324	AGT98514.1	https://doi.org/10.1128%2FAAC.00477-13
NWT	*C. tropicalis*	Yeast	ERG11	KC542325	AGT98515.1	https://doi.org/10.1128%2FAAC.00477-13
NWT	*C. tropicalis*	Yeast	ERG11	KC542326	AGT98516.1	https://doi.org/10.1128%2FAAC.00477-13
WT	*C. tropicalis*	Yeast	ERG11	AAX39313.1	AAX39313.1	https://doi.org/10.1128/AAC.49.11.4608-4615.2005
WT	*C. tropicalis*	Yeast	ERG11	AAX39314.1	AAX39314.1	https://doi.org/10.1128/AAC.49.11.4608-4615.2005
NWT	*C. tropicalis*	Yeast	ERG11	AAX39315.1	AAX39315.1	https://doi.org/10.1128/AAC.49.11.4608-4615.2005
WT	*C. tropicalis*	Yeast	ERG11	AAX39316.1	AAX39316.1	https://doi.org/10.1128/AAC.49.11.4608-4615.2005
NWT	*C. glabrata*	Yeast	ERG11	MH734272	QAX24681.1	https://doi.org/10.1016/j.jgar.2019.01.006
NWT	*C. glabrata*	Yeast	ERG11	MH734273	QAX24682.1	https://doi.org/10.1016/j.jgar.2019.01.006
NWT	*C. glabrata*	Yeast	ERG11	MH734274	QAX24683.1	https://doi.org/10.1016/j.jgar.2019.01.006
NWT	*C. glabrata*	Yeast	ERG11	MH734275	QAX24684.1	https://doi.org/10.1016/j.jgar.2019.01.006
NWT	*C. glabrata*	Yeast	ERG11	MH734276	QAX24685.1	https://doi.org/10.1016/j.jgar.2019.01.006
NWT	*C. glabrata*	Yeast	ERG11	MH734277	QAX24686.1	https://doi.org/10.1016/j.jgar.2019.01.006
WT	*C. glabrata*	Yeast	ERG11	MH734278	QAX24687.1	https://doi.org/10.1016/j.jgar.2019.01.006
WT	*C. glabrata*	Yeast	ERG11	MH734279	QAX24688.1	https://doi.org/10.1016/j.jgar.2019.01.006
WT	*C. glabrata*	Yeast	ERG11	MH734280	QAX24689.1	https://doi.org/10.1016/j.jgar.2019.01.006
WT	*C. glabrata*	Yeast	ERG11	MH734281	QAX24690.1	https://doi.org/10.1016/j.jgar.2019.01.006
WT	*C. glabrata*	Yeast	ERG11	MH734282	QAX24691.1	https://doi.org/10.1016/j.jgar.2019.01.006
NWT	*N. keratoplastica*	Filamentous	CYP51A	MN296712	QGR26263.1	https://doi.org/10.3389/fmicb.2020.00272
NWT	*N. keratoplastica*	Filamentous	CYP51A	MN296713	QGR26264.1	https://doi.org/10.3389/fmicb.2020.00272
NWT	*N. keratoplastica*	Filamentous	CYP51A	MN296714	QGR26265.1	https://doi.org/10.3389/fmicb.2020.00272
NWT	*N. keratoplastica*	Filamentous	CYP51A	MN296715	QGR26266.1	https://doi.org/10.3389/fmicb.2020.00272
NWT	*N. keratoplastica*	Filamentous	CYP51A	MN296716	QGR26267.1	https://doi.org/10.3389/fmicb.2020.00272
NWT	*N. keratoplastica*	Filamentous	CYP51A	MN296717	QGR26268.1	https://doi.org/10.3389/fmicb.2020.00272
NWT	*N. keratoplastica*	Filamentous	CYP51A	MN296718	QGR26269.1	https://doi.org/10.3389/fmicb.2020.00272
NWT	*N. keratoplastica*	Filamentous	CYP51A	MN296719	QGR26270.1	https://doi.org/10.3389/fmicb.2020.00272
NWT	*N. suttoniana*	Filamentous	CYP51A	MN640614	QGZ00345.1	https://doi.org/10.3389/fmicb.2020.00272
NWT	*N. suttoniana*	Filamentous	CYP51A	MN640615	QGZ00346.1	https://doi.org/10.3389/fmicb.2020.00272
NWT	*N. suttoniana*	Filamentous	CYP51A	MN640617	QGZ00347.1	https://doi.org/10.3389/fmicb.2020.00272
NWT	*N. suttoniana*	Filamentous	CYP51A	MN640618	QGZ00348.1	https://doi.org/10.3389/fmicb.2020.00272
NWT	*N. falciformis*	Filamentous	CYP51B	MN640620	QGZ00349.1	https://doi.org/10.3389/fmicb.2020.00272
NWT	*N. falciformis*	Filamentous	CYP51B	MN640621	QGZ00350.1	https://doi.org/10.3389/fmicb.2020.00272
NWT	*N. keratoplastica*	Filamentous	CYP51B	MN296722	QGR26271.1	https://doi.org/10.3389/fmicb.2020.00272
NWT	*N. keratoplastica*	Filamentous	CYP51B	MN296723	QGR26272.1	https://doi.org/10.3389/fmicb.2020.00272
NWT	*N. keratoplastica*	Filamentous	CYP51B	MN296724	QGR26273.1	https://doi.org/10.3389/fmicb.2020.00272
NWT	*N. keratoplastica*	Filamentous	CYP51C	MN296725	QGR26274.1	https://doi.org/10.3389/fmicb.2020.00272
NWT	*F. keratoplasticum*	Filamentous	CYP51A	Supplementary material table S1 of the original paper – named as FK0001.1	MLSLVSYPVWAIA	https://doi.org/10.3390/jof8101070
NWT	*F. keratoplasticum*	Filamentous	CYP51B	Supplementary material table S1 of the original paper – named ad FK0002.1	MGLLHEIAGHPLA	https://doi.org/10.3390/jof8101070
NWT	*F. keratoplasticum*	Filamentous	CYP51C	Supplementary material table S1 of the original paper – named as FK0003.1	MESLYGSFRALPL	https://doi.org/10.3390/jof8101070
WT	*A. fumigatus*		CYP51	Annotated in this study	ERR769498	https://doi.org/10.1128/mbio.00536-15
WT	*A. fumigatus*		CYP51	Annotated in this study	ERR769498_2	https://doi.org/10.1128/mbio.00536-15
NWT	*A. fumigatus*		CYP51	Annotated in this study	ERR769499	https://doi.org/10.1128/mbio.00536-15
NWT	*A. fumigatus*		CYP51	Annotated in this study	ERR769499_2	https://doi.org/10.1128/mbio.00536-15
NWT	*A. fumigatus*		CYP51	Annotated in this study	ERR769500	https://doi.org/10.1128/mbio.00536-15
NWT	*A. fumigatus*		CYP51	Annotated in this study	ERR769500_2	https://doi.org/10.1128/mbio.00536-15
NWT	*A. fumigatus*		CYP51	Annotated in this study	ERR769501	https://doi.org/10.1128/mbio.00536-15
NWT	*A. fumigatus*		CYP51	Annotated in this study	ERR769501_2	https://doi.org/10.1128/mbio.00536-15

NWT	*A. fumigatus*	CYP51	Annotated in this study	ERR769503	https://doi.org/10.1128/mbio.00536-15
NWT	*A. fumigatus*	CYP51	Annotated in this study	ERR769503_2	https://doi.org/10.1128/mbio.00536-15
NWT	*A. fumigatus*	CYP51	Annotated in this study	ERR769502	https://doi.org/10.1128/mbio.00536-15
NWT	*A. fumigatus*	CYP51	Annotated in this study	ERR769502_2	https://doi.org/10.1128/mbio.00536-15
NWT	*A. fumigatus*	CYP51	Annotated in this study	ERR769504	https://doi.org/10.1128/mbio.00536-15
NWT	*A. fumigatus*	CYP51	Annotated in this study	ERR769504_2	https://doi.org/10.1128/mbio.00536-15
NWT	*A. fumigatus*	CYP51	Annotated in this study	ERR769506	https://doi.org/10.1128/mbio.00536-15
NWT	*A. fumigatus*	CYP51	Annotated in this study	ERR769506_2	https://doi.org/10.1128/mbio.00536-15
NWT	*A. fumigatus*	CYP51	Annotated in this study	ERR769505	https://doi.org/10.1128/mbio.00536-15
NWT	*A. fumigatus*	CYP51	Annotated in this study	ERR769505_2	https://doi.org/10.1128/mbio.00536-15
NWT	*A. fumigatus*	CYP51	Annotated in this study	ERR769507	https://doi.org/10.1128/mbio.00536-15
NWT	*A. fumigatus*	CYP51	Annotated in this study	ERR769507_2	https://doi.org/10.1128/mbio.00536-15
NWT	*A. fumigatus*	CYP51	Annotated in this study	ERR769508	https://doi.org/10.1128/mbio.00536-15
NWT	*A. fumigatus*	CYP51	Annotated in this study	ERR769508_2	https://doi.org/10.1128/mbio.00536-15
NWT	*A. fumigatus*	CYP51	Annotated in this study	ERR769509	https://doi.org/10.1128/mbio.00536-15
NWT	*A. fumigatus*	CYP51	Annotated in this study	ERR769509_2	https://doi.org/10.1128/mbio.00536-15
WT	*A. fumigatus*	CYP51	Annotated in this study	ERR769510	https://doi.org/10.1128/mbio.00536-15
WT	*A. fumigatus*	CYP51	Annotated in this study	ERR769510_2	https://doi.org/10.1128/mbio.00536-15
NWT	*A. fumigatus*	CYP51	Annotated in this study	ERR769511	https://doi.org/10.1128/mbio.00536-15
NWT	*A. fumigatus*	CYP51	Annotated in this study	ERR769511_2	https://doi.org/10.1128/mbio.00536-15
NWT	*A. fumigatus*	CYP51	Annotated in this study	ERR769512	https://doi.org/10.1128/mbio.00536-15
NWT	*A. fumigatus*	CYP51	Annotated in this study	ERR769512_2	https://doi.org/10.1128/mbio.00536-15
NWT	*A. fumigatus*	CYP51	Annotated in this study	ERR769513	https://doi.org/10.1128/mbio.00536-15
NWT	*A. fumigatus*	CYP51	Annotated in this study	ERR769513_2	https://doi.org/10.1128/mbio.00536-15
NWT	*A. fumigatus*	CYP51	Annotated in this study	ERR769514	https://doi.org/10.1128/mbio.00536-15
NWT	*A. fumigatus*	CYP51	Annotated in this study	ERR769514_2	https://doi.org/10.1128/mbio.00536-15
NWT	*A. fumigatus*	CYP51	Annotated in this study	ERR769515	https://doi.org/10.1128/mbio.00536-15
NWT	*A. fumigatus*	CYP51	Annotated in this study	ERR769515_2	https://doi.org/10.1128/mbio.00536-15
NWT	*A. fumigatus*	CYP51	Annotated in this study	ERR769516	https://doi.org/10.1128/mbio.00536-15
NWT	*A. fumigatus*	CYP51	Annotated in this study	ERR769516_2	https://doi.org/10.1128/mbio.00536-15
NWT	*A. fumigatus*	CYP51	Annotated in this study	ERR769517	https://doi.org/10.1128/mbio.00536-15
NWT	*A. fumigatus*	CYP51	Annotated in this study	ERR769517_2	https://doi.org/10.1128/mbio.00536-15
NWT	*A. fumigatus*	CYP51	Annotated in this study	ERR769518	https://doi.org/10.1128/mbio.00536-15
NWT	*A. fumigatus*	CYP51	Annotated in this study	ERR769518_2	https://doi.org/10.1128/mbio.00536-15
NWT	*A. fumigatus*	CYP51	Annotated in this study	ERR769519	https://doi.org/10.1128/mbio.00536-15
NWT	*A. fumigatus*	CYP51	Annotated in this study	ERR769519_2	https://doi.org/10.1128/mbio.00536-15
NWT	*A. fumigatus*	CYP51	Annotated in this study	ERR769520	https://doi.org/10.1128/mbio.00536-15
NWT	*A. fumigatus*	CYP51	Annotated in this study	ERR769520_2	https://doi.org/10.1128/mbio.00536-15
NWT	*A. fumigatus*	CYP51	Annotated in this study	ERR769521	https://doi.org/10.1128/mbio.00536-15
NWT	*A. fumigatus*	CYP51	Annotated in this study	ERR769521_2	https://doi.org/10.1128/mbio.00536-15
NWT	*A. fumigatus*	CYP51	Annotated in this study	ERR9791571	https://doi.org/10.1038/s41564-022-01091-2
NWT	*A. fumigatus*	CYP51	Annotated in this study	ERR9791571_2	https://doi.org/10.1038/s41564-022-01091-2
NWT	*A. fumigatus*	CYP51	Annotated in this study	ERR9791572	https://doi.org/10.1038/s41564-022-01091-2
NWT	*A. fumigatus*	CYP51	Annotated in this study	ERR9791572_2	https://doi.org/10.1038/s41564-022-01091-2
NWT	*A. fumigatus*	CYP51	Annotated in this study	ERR9791573	https://doi.org/10.1038/s41564-022-01091-2
NWT	*A. fumigatus*	CYP51	Annotated in this study	ERR9791573_2	https://doi.org/10.1038/s41564-022-01091-2
WT	*A. fumigatus*	CYP51	Annotated in this study	ERR9791574	https://doi.org/10.1038/s41564-022-01091-2
WT	*A. fumigatus*	CYP51	Annotated in this study	ERR9791574_2	https://doi.org/10.1038/s41564-022-01091-2
NWT	*A. fumigatus*	CYP51	Annotated in this study	ERR9791576	https://doi.org/10.1038/s41564-022-01091-2

NWT	*A. fumigatus*	CYP51	Annotated in this study	ERR9791576_2	https://doi.org/10.1038/s41564-022-01091-2
NWT	*A. fumigatus*	CYP51	Annotated in this study	ERR9791577	https://doi.org/10.1038/s41564-022-01091-2
NWT	*A. fumigatus*	CYP51	Annotated in this study	ERR9791577_2	https://doi.org/10.1038/s41564-022-01091-2
NWT	*A. fumigatus*	CYP51	Annotated in this study	ERR9791575	https://doi.org/10.1038/s41564-022-01091-2
NWT	*A. fumigatus*	CYP51	Annotated in this study	ERR9791575_2	https://doi.org/10.1038/s41564-022-01091-2
NWT	*A. fumigatus*	CYP51	Annotated in this study	ERR9791578	https://doi.org/10.1038/s41564-022-01091-2
NWT	*A. fumigatus*	CYP51	Annotated in this study	ERR9791578_2	https://doi.org/10.1038/s41564-022-01091-2
NWT	*A. fumigatus*	CYP51	Annotated in this study	ERR9791579	https://doi.org/10.1038/s41564-022-01091-2
NWT	*A. fumigatus*	CYP51	Annotated in this study	ERR9791579_2	https://doi.org/10.1038/s41564-022-01091-2
NWT	*A. fumigatus*	CYP51	Annotated in this study	ERR9791580	https://doi.org/10.1038/s41564-022-01091-2
NWT	*A. fumigatus*	CYP51	Annotated in this study	ERR9791580_2	https://doi.org/10.1038/s41564-022-01091-2
NWT	*A. fumigatus*	CYP51	Annotated in this study	ERR9791581	https://doi.org/10.1038/s41564-022-01091-2
NWT	*A. fumigatus*	CYP51	Annotated in this study	ERR9791581_2	https://doi.org/10.1038/s41564-022-01091-2
NWT	*A. fumigatus*	CYP51	Annotated in this study	ERR9791582	https://doi.org/10.1038/s41564-022-01091-2
NWT	*A. fumigatus*	CYP51	Annotated in this study	ERR9791582_2	https://doi.org/10.1038/s41564-022-01091-2
NWT	*A. fumigatus*	CYP51	Annotated in this study	ERR9791583	https://doi.org/10.1038/s41564-022-01091-2
NWT	*A. fumigatus*	CYP51	Annotated in this study	ERR9791583_2	https://doi.org/10.1038/s41564-022-01091-2
NWT	*A. fumigatus*	CYP51	Annotated in this study	ERR9791585	https://doi.org/10.1038/s41564-022-01091-2
NWT	*A. fumigatus*	CYP51	Annotated in this study	ERR9791585_2	https://doi.org/10.1038/s41564-022-01091-2
NWT	*A. fumigatus*	CYP51	Annotated in this study	ERR9791585_3	https://doi.org/10.1038/s41564-022-01091-2
NWT	*A. fumigatus*	CYP51	Annotated in this study	ERR9791584	https://doi.org/10.1038/s41564-022-01091-2
NWT	*A. fumigatus*	CYP51	Annotated in this study	ERR9791584_2	https://doi.org/10.1038/s41564-022-01091-2
NWT	*A. fumigatus*	CYP51	Annotated in this study	ERR9791586	https://doi.org/10.1038/s41564-022-01091-2
NWT	*A. fumigatus*	CYP51	Annotated in this study	ERR9791586_2	https://doi.org/10.1038/s41564-022-01091-2
NWT	*A. fumigatus*	CYP51	Annotated in this study	ERR9791587	https://doi.org/10.1038/s41564-022-01091-2
NWT	*A. fumigatus*	CYP51	Annotated in this study	ERR9791587_2	https://doi.org/10.1038/s41564-022-01091-2
NWT	*A. fumigatus*	CYP51	Annotated in this study	ERR9791588	https://doi.org/10.1038/s41564-022-01091-2
NWT	*A. fumigatus*	CYP51	Annotated in this study	ERR9791588_2	https://doi.org/10.1038/s41564-022-01091-2
NWT	*A. fumigatus*	CYP51	Annotated in this study	ERR9791590	https://doi.org/10.1038/s41564-022-01091-2
NWT	*A. fumigatus*	CYP51	Annotated in this study	ERR9791590_2	https://doi.org/10.1038/s41564-022-01091-2
NWT	*A. fumigatus*	CYP51	Annotated in this study	ERR9791591	https://doi.org/10.1038/s41564-022-01091-2
NWT	*A. fumigatus*	CYP51	Annotated in this study	ERR9791591_2	https://doi.org/10.1038/s41564-022-01091-2
NWT	*A. fumigatus*	CYP51	Annotated in this study	ERR9791592	https://doi.org/10.1038/s41564-022-01091-2
NWT	*A. fumigatus*	CYP51	Annotated in this study	ERR9791592_2	https://doi.org/10.1038/s41564-022-01091-2
NWT	*A. fumigatus*	CYP51	Annotated in this study	ERR9791589	https://doi.org/10.1038/s41564-022-01091-2
NWT	*A. fumigatus*	CYP51	Annotated in this study	ERR9791589_2	https://doi.org/10.1038/s41564-022-01091-2
NWT	*A. fumigatus*	CYP51	Annotated in this study	ERR9791593	https://doi.org/10.1038/s41564-022-01091-2
NWT	*A. fumigatus*	CYP51	Annotated in this study	ERR9791593_2	https://doi.org/10.1038/s41564-022-01091-2
NWT	*A. fumigatus*	CYP51	Annotated in this study	ERR9791594	https://doi.org/10.1038/s41564-022-01091-2
NWT	*A. fumigatus*	CYP51	Annotated in this study	ERR9791594_2	https://doi.org/10.1038/s41564-022-01091-2
NWT	*A. fumigatus*	CYP51	Annotated in this study	ERR9791595	https://doi.org/10.1038/s41564-022-01091-2
NWT	*A. fumigatus*	CYP51	Annotated in this study	ERR9791595_2	https://doi.org/10.1038/s41564-022-01091-2
NWT	*A. fumigatus*	CYP51	Annotated in this study	ERR9791596	https://doi.org/10.1038/s41564-022-01091-2
NWT	*A. fumigatus*	CYP51	Annotated in this study	ERR9791596_2	https://doi.org/10.1038/s41564-022-01091-2
NWT	*A. fumigatus*	CYP51	Annotated in this study	ERR9791597	https://doi.org/10.1038/s41564-022-01091-2
NWT	*A. fumigatus*	CYP51	Annotated in this study	ERR9791597_2	https://doi.org/10.1038/s41564-022-01091-2
NWT	*A. fumigatus*	CYP51	Annotated in this study	ERR9791598	https://doi.org/10.1038/s41564-022-01091-2
NWT	*A. fumigatus*	CYP51	Annotated in this study	ERR9791598_2	https://doi.org/10.1038/s41564-022-01091-2
NWT	*A. fumigatus*	CYP51	Annotated in this study	ERR9791599	https://doi.org/10.1038/s41564-022-01091-2

NWT	*A. fumigatus*	CYP51	Annotated in this study	ERR9791599_2	https://doi.org/10.1038/s41564-022-01091-2
NWT	*A. fumigatus*	CYP51	Annotated in this study	ERR9791600	https://doi.org/10.1038/s41564-022-01091-2
NWT	*A. fumigatus*	CYP51	Annotated in this study	ERR9791600_2	https://doi.org/10.1038/s41564-022-01091-2
NWT	*A. fumigatus*	CYP51	Annotated in this study	ERR9791601	https://doi.org/10.1038/s41564-022-01091-2
NWT	*A. fumigatus*	CYP51	Annotated in this study	ERR9791601_2	https://doi.org/10.1038/s41564-022-01091-2
NWT	*A. fumigatus*	CYP51	Annotated in this study	ERR9791602	https://doi.org/10.1038/s41564-022-01091-2
NWT	*A. fumigatus*	CYP51	Annotated in this study	ERR9791602_2	https://doi.org/10.1038/s41564-022-01091-2
WT	*A. fumigatus*	CYP51	Annotated in this study	ERR9791603	https://doi.org/10.1038/s41564-022-01091-2
WT	*A. fumigatus*	CYP51	Annotated in this study	ERR9791603_2	https://doi.org/10.1038/s41564-022-01091-2
WT	*A. fumigatus*	CYP51	Annotated in this study	ERR9791604	https://doi.org/10.1038/s41564-022-01091-2
WT	*A. fumigatus*	CYP51	Annotated in this study	ERR9791604_2	https://doi.org/10.1038/s41564-022-01091-2
WT	*A. fumigatus*	CYP51	Annotated in this study	ERR9791605	https://doi.org/10.1038/s41564-022-01091-2
WT	*A. fumigatus*	CYP51	Annotated in this study	ERR9791605_2	https://doi.org/10.1038/s41564-022-01091-2
WT	*A. fumigatus*	CYP51	Annotated in this study	ERR9791606	https://doi.org/10.1038/s41564-022-01091-2
WT	*A. fumigatus*	CYP51	Annotated in this study	ERR9791606_2	https://doi.org/10.1038/s41564-022-01091-2
WT	*A. fumigatus*	CYP51	Annotated in this study	ERR9791607	https://doi.org/10.1038/s41564-022-01091-2
WT	*A. fumigatus*	CYP51	Annotated in this study	ERR9791607_2	https://doi.org/10.1038/s41564-022-01091-2
WT	*A. fumigatus*	CYP51	Annotated in this study	ERR9791608	https://doi.org/10.1038/s41564-022-01091-2
WT	*A. fumigatus*	CYP51	Annotated in this study	ERR9791608_2	https://doi.org/10.1038/s41564-022-01091-2
WT	*A. fumigatus*	CYP51	Annotated in this study	ERR9791611	https://doi.org/10.1038/s41564-022-01091-2
WT	*A. fumigatus*	CYP51	Annotated in this study	ERR9791611_2	https://doi.org/10.1038/s41564-022-01091-2
WT	*A. fumigatus*	CYP51	Annotated in this study	ERR9791612	https://doi.org/10.1038/s41564-022-01091-2
WT	*A. fumigatus*	CYP51	Annotated in this study	ERR9791612_2	https://doi.org/10.1038/s41564-022-01091-2
WT	*A. fumigatus*	CYP51	Annotated in this study	ERR9791609	https://doi.org/10.1038/s41564-022-01091-2
WT	*A. fumigatus*	CYP51	Annotated in this study	ERR9791609_2	https://doi.org/10.1038/s41564-022-01091-2
WT	*A. fumigatus*	CYP51	Annotated in this study	ERR9791613	https://doi.org/10.1038/s41564-022-01091-2
WT	*A. fumigatus*	CYP51	Annotated in this study	ERR9791613_2	https://doi.org/10.1038/s41564-022-01091-2
WT	*A. fumigatus*	CYP51	Annotated in this study	ERR9791610	https://doi.org/10.1038/s41564-022-01091-2
WT	*A. fumigatus*	CYP51	Annotated in this study	ERR9791610_2	https://doi.org/10.1038/s41564-022-01091-2
WT	*A. fumigatus*	CYP51	Annotated in this study	ERR9791614	https://doi.org/10.1038/s41564-022-01091-2
WT	*A. fumigatus*	CYP51	Annotated in this study	ERR9791614_2	https://doi.org/10.1038/s41564-022-01091-2
WT	*A. fumigatus*	CYP51	Annotated in this study	ERR9791615	https://doi.org/10.1038/s41564-022-01091-2
WT	*A. fumigatus*	CYP51	Annotated in this study	ERR9791615_2	https://doi.org/10.1038/s41564-022-01091-2
WT	*A. fumigatus*	CYP51	Annotated in this study	ERR9791618	https://doi.org/10.1038/s41564-022-01091-2
WT	*A. fumigatus*	CYP51	Annotated in this study	ERR9791618_2	https://doi.org/10.1038/s41564-022-01091-2
WT	*A. fumigatus*	CYP51	Annotated in this study	ERR9791619	https://doi.org/10.1038/s41564-022-01091-2
WT	*A. fumigatus*	CYP51	Annotated in this study	ERR9791619_2	https://doi.org/10.1038/s41564-022-01091-2
WT	*A. fumigatus*	CYP51	Annotated in this study	ERR9791616	https://doi.org/10.1038/s41564-022-01091-2
WT	*A. fumigatus*	CYP51	Annotated in this study	ERR9791616_2	https://doi.org/10.1038/s41564-022-01091-2
WT	*A. fumigatus*	CYP51	Annotated in this study	ERR9791620	https://doi.org/10.1038/s41564-022-01091-2
WT	*A. fumigatus*	CYP51	Annotated in this study	ERR9791620_2	https://doi.org/10.1038/s41564-022-01091-2
WT	*A. fumigatus*	CYP51	Annotated in this study	ERR9791617	https://doi.org/10.1038/s41564-022-01091-2
WT	*A. fumigatus*	CYP51	Annotated in this study	ERR9791617_2	https://doi.org/10.1038/s41564-022-01091-2
WT	*A. fumigatus*	CYP51	Annotated in this study	ERR9791621	https://doi.org/10.1038/s41564-022-01091-2
WT	*A. fumigatus*	CYP51	Annotated in this study	ERR9791621_2	https://doi.org/10.1038/s41564-022-01091-2
WT	*A. fumigatus*	CYP51	Annotated in this study	ERR9791622	https://doi.org/10.1038/s41564-022-01091-2
WT	*A. fumigatus*	CYP51	Annotated in this study	ERR9791622_2	https://doi.org/10.1038/s41564-022-01091-2
WT	*A. fumigatus*	CYP51	Annotated in this study	ERR9791624	https://doi.org/10.1038/s41564-022-01091-2
WT	*A. fumigatus*	CYP51	Annotated in this study	ERR9791624_2	https://doi.org/10.1038/s41564-022-01091-2

WT	*A. fumigatus*	CYP51	Annotated in this study	ERR9791623	https://doi.org/10.1038/s41564-022-01091-2
WT	*A. fumigatus*	CYP51	Annotated in this study	ERR9791623_2	https://doi.org/10.1038/s41564-022-01091-2
WT	*A. fumigatus*	CYP51	Annotated in this study	ERR9791625	https://doi.org/10.1038/s41564-022-01091-2
WT	*A. fumigatus*	CYP51	Annotated in this study	ERR9791625_2	https://doi.org/10.1038/s41564-022-01091-2
WT	*A. fumigatus*	CYP51	Annotated in this study	ERR9791626	https://doi.org/10.1038/s41564-022-01091-2
WT	*A. fumigatus*	CYP51	Annotated in this study	ERR9791626_2	https://doi.org/10.1038/s41564-022-01091-2
WT	*A. fumigatus*	CYP51	Annotated in this study	ERR9791629	https://doi.org/10.1038/s41564-022-01091-2
WT	*A. fumigatus*	CYP51	Annotated in this study	ERR9791629_2	https://doi.org/10.1038/s41564-022-01091-2
WT	*A. fumigatus*	CYP51	Annotated in this study	ERR9791627	https://doi.org/10.1038/s41564-022-01091-2
WT	*A. fumigatus*	CYP51	Annotated in this study	ERR9791627_2	https://doi.org/10.1038/s41564-022-01091-2
WT	*A. fumigatus*	CYP51	Annotated in this study	ERR9791628	https://doi.org/10.1038/s41564-022-01091-2
WT	*A. fumigatus*	CYP51	Annotated in this study	ERR9791628_2	https://doi.org/10.1038/s41564-022-01091-2
WT	*A. fumigatus*	CYP51	Annotated in this study	ERR9791630	https://doi.org/10.1038/s41564-022-01091-2
WT	*A. fumigatus*	CYP51	Annotated in this study	ERR9791630_2	https://doi.org/10.1038/s41564-022-01091-2
WT	*A. fumigatus*	CYP51	Annotated in this study	ERR9791633	https://doi.org/10.1038/s41564-022-01091-2
WT	*A. fumigatus*	CYP51	Annotated in this study	ERR9791633_2	https://doi.org/10.1038/s41564-022-01091-2
WT	*A. fumigatus*	CYP51	Annotated in this study	ERR9791631	https://doi.org/10.1038/s41564-022-01091-2
WT	*A. fumigatus*	CYP51	Annotated in this study	ERR9791631_2	https://doi.org/10.1038/s41564-022-01091-2
WT	*A. fumigatus*	CYP51	Annotated in this study	ERR9791632	https://doi.org/10.1038/s41564-022-01091-2
WT	*A. fumigatus*	CYP51	Annotated in this study	ERR9791632_2	https://doi.org/10.1038/s41564-022-01091-2
WT	*A. fumigatus*	CYP51	Annotated in this study	ERR9791634	https://doi.org/10.1038/s41564-022-01091-2
WT	*A. fumigatus*	CYP51	Annotated in this study	ERR9791634_2	https://doi.org/10.1038/s41564-022-01091-2
WT	*A. fumigatus*	CYP51	Annotated in this study	ERR9791635	https://doi.org/10.1038/s41564-022-01091-2
WT	*A. fumigatus*	CYP51	Annotated in this study	ERR9791635_2	https://doi.org/10.1038/s41564-022-01091-2
WT	*A. fumigatus*	CYP51	Annotated in this study	ERR9791636	https://doi.org/10.1038/s41564-022-01091-2
WT	*A. fumigatus*	CYP51	Annotated in this study	ERR9791636_2	https://doi.org/10.1038/s41564-022-01091-2
NWT	*A. fumigatus*	CYP51	Annotated in this study	ERR9791637	https://doi.org/10.1038/s41564-022-01091-2
NWT	*A. fumigatus*	CYP51	Annotated in this study	ERR9791637_2	https://doi.org/10.1038/s41564-022-01091-2
NWT	*A. fumigatus*	CYP51	Annotated in this study	ERR9791638	https://doi.org/10.1038/s41564-022-01091-2
NWT	*A. fumigatus*	CYP51	Annotated in this study	ERR9791638_2	https://doi.org/10.1038/s41564-022-01091-2
NWT	*A. fumigatus*	CYP51	Annotated in this study	ERR9791639	https://doi.org/10.1038/s41564-022-01091-2
NWT	*A. fumigatus*	CYP51	Annotated in this study	ERR9791639_2	https://doi.org/10.1038/s41564-022-01091-2
WT	*A. fumigatus*	CYP51	Annotated in this study	ERR9791640	https://doi.org/10.1038/s41564-022-01091-2
WT	*A. fumigatus*	CYP51	Annotated in this study	ERR9791640_2	https://doi.org/10.1038/s41564-022-01091-2
WT	*A. fumigatus*	CYP51	Annotated in this study	ERR9791641	https://doi.org/10.1038/s41564-022-01091-2
WT	*A. fumigatus*	CYP51	Annotated in this study	ERR9791641_2	https://doi.org/10.1038/s41564-022-01091-2
WT	*A. fumigatus*	CYP51	Annotated in this study	ERR9791642	https://doi.org/10.1038/s41564-022-01091-2
WT	*A. fumigatus*	CYP51	Annotated in this study	ERR9791642_2	https://doi.org/10.1038/s41564-022-01091-2
WT	*A. fumigatus*	CYP51	Annotated in this study	ERR9791643	https://doi.org/10.1038/s41564-022-01091-2
WT	*A. fumigatus*	CYP51	Annotated in this study	ERR9791643_2	https://doi.org/10.1038/s41564-022-01091-2
WT	*A. fumigatus*	CYP51	Annotated in this study	ERR9791645	https://doi.org/10.1038/s41564-022-01091-2
WT	*A. fumigatus*	CYP51	Annotated in this study	ERR9791645_2	https://doi.org/10.1038/s41564-022-01091-2
WT	*A. fumigatus*	CYP51	Annotated in this study	ERR9791644	https://doi.org/10.1038/s41564-022-01091-2
WT	*A. fumigatus*	CYP51	Annotated in this study	ERR9791644_2	https://doi.org/10.1038/s41564-022-01091-2
WT	*A. fumigatus*	CYP51	Annotated in this study	ERR9791646	https://doi.org/10.1038/s41564-022-01091-2
WT	*A. fumigatus*	CYP51	Annotated in this study	ERR9791646_2	https://doi.org/10.1038/s41564-022-01091-2
NWT	*A. fumigatus*	CYP51	Annotated in this study	ERR9791647	https://doi.org/10.1038/s41564-022-01091-2
NWT	*A. fumigatus*	CYP51	Annotated in this study	ERR9791647_2	https://doi.org/10.1038/s41564-022-01091-2
NWT	*A. fumigatus*	CYP51	Annotated in this study	ERR9791648	https://doi.org/10.1038/s41564-022-01091-2

NWT	*A. fumigatus*	CYP51	Annotated in this study	ERR9791648_2	https://doi.org/10.1038/s41564-022-01091-2
WT	*A. fumigatus*	CYP51	Annotated in this study	ERR9791650	https://doi.org/10.1038/s41564-022-01091-2
WT	*A. fumigatus*	CYP51	Annotated in this study	ERR9791650_2	https://doi.org/10.1038/s41564-022-01091-2
NWT	*A. fumigatus*	CYP51	Annotated in this study	ERR9791649	https://doi.org/10.1038/s41564-022-01091-2
NWT	*A. fumigatus*	CYP51	Annotated in this study	ERR9791649_2	https://doi.org/10.1038/s41564-022-01091-2
NWT	*A. fumigatus*	CYP51	Annotated in this study	ERR9791651	https://doi.org/10.1038/s41564-022-01091-2
NWT	*A. fumigatus*	CYP51	Annotated in this study	ERR9791651_2	https://doi.org/10.1038/s41564-022-01091-2
NWT	*A. fumigatus*	CYP51	Annotated in this study	ERR9791652	https://doi.org/10.1038/s41564-022-01091-2
NWT	*A. fumigatus*	CYP51	Annotated in this study	ERR9791652_2	https://doi.org/10.1038/s41564-022-01091-2
NWT	*A. fumigatus*	CYP51	Annotated in this study	ERR9791653	https://doi.org/10.1038/s41564-022-01091-2
NWT	*A. fumigatus*	CYP51	Annotated in this study	ERR9791653_2	https://doi.org/10.1038/s41564-022-01091-2
NWT	*A. fumigatus*	CYP51	Annotated in this study	ERR9791654	https://doi.org/10.1038/s41564-022-01091-2
NWT	*A. fumigatus*	CYP51	Annotated in this study	ERR9791654_2	https://doi.org/10.1038/s41564-022-01091-2
NWT	*A. fumigatus*	CYP51	Annotated in this study	ERR9791655	https://doi.org/10.1038/s41564-022-01091-2
NWT	*A. fumigatus*	CYP51	Annotated in this study	ERR9791655_2	https://doi.org/10.1038/s41564-022-01091-2
NWT	*A. fumigatus*	CYP51	Annotated in this study	ERR9791656	https://doi.org/10.1038/s41564-022-01091-2
NWT	*A. fumigatus*	CYP51	Annotated in this study	ERR9791656_2	https://doi.org/10.1038/s41564-022-01091-2
NWT	*A. fumigatus*	CYP51	Annotated in this study	ERR9791657	https://doi.org/10.1038/s41564-022-01091-2
NWT	*A. fumigatus*	CYP51	Annotated in this study	ERR9791657_2	https://doi.org/10.1038/s41564-022-01091-2
NWT	*A. fumigatus*	CYP51	Annotated in this study	ERR9791658	https://doi.org/10.1038/s41564-022-01091-2
NWT	*A. fumigatus*	CYP51	Annotated in this study	ERR9791658_2	https://doi.org/10.1038/s41564-022-01091-2
NWT	*A. fumigatus*	CYP51	Annotated in this study	ERR9791661	https://doi.org/10.1038/s41564-022-01091-2
NWT	*A. fumigatus*	CYP51	Annotated in this study	ERR9791661_2	https://doi.org/10.1038/s41564-022-01091-2
NWT	*A. fumigatus*	CYP51	Annotated in this study	ERR9791659	https://doi.org/10.1038/s41564-022-01091-2
NWT	*A. fumigatus*	CYP51	Annotated in this study	ERR9791659_2	https://doi.org/10.1038/s41564-022-01091-2
WT	*A. fumigatus*	CYP51	Annotated in this study	ERR9791662	https://doi.org/10.1038/s41564-022-01091-2
WT	*A. fumigatus*	CYP51	Annotated in this study	ERR9791662_2	https://doi.org/10.1038/s41564-022-01091-2
NWT	*A. fumigatus*	CYP51	Annotated in this study	ERR9791660	https://doi.org/10.1038/s41564-022-01091-2
NWT	*A. fumigatus*	CYP51	Annotated in this study	ERR9791660_2	https://doi.org/10.1038/s41564-022-01091-2
NWT	*A. fumigatus*	CYP51	Annotated in this study	ERR9791664	https://doi.org/10.1038/s41564-022-01091-2
NWT	*A. fumigatus*	CYP51	Annotated in this study	ERR9791664_2	https://doi.org/10.1038/s41564-022-01091-2
WT	*A. fumigatus*	CYP51	Annotated in this study	ERR9791663	https://doi.org/10.1038/s41564-022-01091-2
WT	*A. fumigatus*	CYP51	Annotated in this study	ERR9791663_2	https://doi.org/10.1038/s41564-022-01091-2
NWT	*A. fumigatus*	CYP51	Annotated in this study	ERR9791665	https://doi.org/10.1038/s41564-022-01091-2
NWT	*A. fumigatus*	CYP51	Annotated in this study	ERR9791665_2	https://doi.org/10.1038/s41564-022-01091-2
NWT	*A. fumigatus*	CYP51	Annotated in this study	ERR9791666	https://doi.org/10.1038/s41564-022-01091-2
NWT	*A. fumigatus*	CYP51	Annotated in this study	ERR9791666_2	https://doi.org/10.1038/s41564-022-01091-2
NWT	*A. fumigatus*	CYP51	Annotated in this study	ERR9791667	https://doi.org/10.1038/s41564-022-01091-2
NWT	*A. fumigatus*	CYP51	Annotated in this study	ERR9791667_2	https://doi.org/10.1038/s41564-022-01091-2
NWT	*A. fumigatus*	CYP51	Annotated in this study	ERR9791668	https://doi.org/10.1038/s41564-022-01091-2
NWT	*A. fumigatus*	CYP51	Annotated in this study	ERR9791668_2	https://doi.org/10.1038/s41564-022-01091-2
NWT	*A. fumigatus*	CYP51	Annotated in this study	ERR9791669	https://doi.org/10.1038/s41564-022-01091-2
NWT	*A. fumigatus*	CYP51	Annotated in this study	ERR9791669_2	https://doi.org/10.1038/s41564-022-01091-2
NWT	*A. fumigatus*	CYP51	Annotated in this study	ERR9791670	https://doi.org/10.1038/s41564-022-01091-2
NWT	*A. fumigatus*	CYP51	Annotated in this study	ERR9791670_2	https://doi.org/10.1038/s41564-022-01091-2
NWT	*A. fumigatus*	CYP51	Annotated in this study	ERR9791671	https://doi.org/10.1038/s41564-022-01091-2
NWT	*A. fumigatus*	CYP51	Annotated in this study	ERR9791671_2	https://doi.org/10.1038/s41564-022-01091-2
NWT	*A. fumigatus*	CYP51	Annotated in this study	ERR9791672	https://doi.org/10.1038/s41564-022-01091-2
NWT	*A. fumigatus*	CYP51	Annotated in this study	ERR9791672_2	https://doi.org/10.1038/s41564-022-01091-2

NWT	*A. fumigatus*	CYP51	Annotated in this study	ERR9791673	https://doi.org/10.1038/s41564-022-01091-2
NWT	*A. fumigatus*	CYP51	Annotated in this study	ERR9791673_2	https://doi.org/10.1038/s41564-022-01091-2
NWT	*A. fumigatus*	CYP51	Annotated in this study	ERR9791674	https://doi.org/10.1038/s41564-022-01091-2
NWT	*A. fumigatus*	CYP51	Annotated in this study	ERR9791674_2	https://doi.org/10.1038/s41564-022-01091-2
NWT	*A. fumigatus*	CYP51	Annotated in this study	ERR9791675	https://doi.org/10.1038/s41564-022-01091-2
NWT	*A. fumigatus*	CYP51	Annotated in this study	ERR9791675_2	https://doi.org/10.1038/s41564-022-01091-2
NWT	*A. fumigatus*	CYP51	Annotated in this study	ERR9791677	https://doi.org/10.1038/s41564-022-01091-2
NWT	*A. fumigatus*	CYP51	Annotated in this study	ERR9791677_2	https://doi.org/10.1038/s41564-022-01091-2
WT	*A. fumigatus*	CYP51	Annotated in this study	ERR9791676	https://doi.org/10.1038/s41564-022-01091-2
WT	*A. fumigatus*	CYP51	Annotated in this study	ERR9791676_2	https://doi.org/10.1038/s41564-022-01091-2
NWT	*A. fumigatus*	CYP51	Annotated in this study	ERR9791678	https://doi.org/10.1038/s41564-022-01091-2
NWT	*A. fumigatus*	CYP51	Annotated in this study	ERR9791678_2	https://doi.org/10.1038/s41564-022-01091-2
WT	*A. fumigatus*	CYP51	Annotated in this study	ERR9791680	https://doi.org/10.1038/s41564-022-01091-2
WT	*A. fumigatus*	CYP51	Annotated in this study	ERR9791680_2	https://doi.org/10.1038/s41564-022-01091-2
WT	*A. fumigatus*	CYP51	Annotated in this study	ERR9791679	https://doi.org/10.1038/s41564-022-01091-2
WT	*A. fumigatus*	CYP51	Annotated in this study	ERR9791679_2	https://doi.org/10.1038/s41564-022-01091-2
NWT	*A. fumigatus*	CYP51	Annotated in this study	ERR9791681	https://doi.org/10.1038/s41564-022-01091-2
NWT	*A. fumigatus*	CYP51	Annotated in this study	ERR9791681_2	https://doi.org/10.1038/s41564-022-01091-2
NWT	*A. fumigatus*	CYP51	Annotated in this study	ERR9791682	https://doi.org/10.1038/s41564-022-01091-2
NWT	*A. fumigatus*	CYP51	Annotated in this study	ERR9791682_2	https://doi.org/10.1038/s41564-022-01091-2
NWT	*A. fumigatus*	CYP51	Annotated in this study	ERR9791683	https://doi.org/10.1038/s41564-022-01091-2
NWT	*A. fumigatus*	CYP51	Annotated in this study	ERR9791683_2	https://doi.org/10.1038/s41564-022-01091-2
NWT	*A. fumigatus*	CYP51	Annotated in this study	ERR9791684	https://doi.org/10.1038/s41564-022-01091-2
NWT	*A. fumigatus*	CYP51	Annotated in this study	ERR9791684_2	https://doi.org/10.1038/s41564-022-01091-2
NWT	*A. fumigatus*	CYP51	Annotated in this study	ERR9791686	https://doi.org/10.1038/s41564-022-01091-2
NWT	*A. fumigatus*	CYP51	Annotated in this study	ERR9791686_2	https://doi.org/10.1038/s41564-022-01091-2
WT	*A. fumigatus*	CYP51	Annotated in this study	ERR9791685	https://doi.org/10.1038/s41564-022-01091-2
WT	*A. fumigatus*	CYP51	Annotated in this study	ERR9791685_2	https://doi.org/10.1038/s41564-022-01091-2
WT	*A. fumigatus*	CYP51	Annotated in this study	ERR9791687	https://doi.org/10.1038/s41564-022-01091-2
WT	*A. fumigatus*	CYP51	Annotated in this study	ERR9791687_2	https://doi.org/10.1038/s41564-022-01091-2
WT	*A. fumigatus*	CYP51	Annotated in this study	ERR9791689	https://doi.org/10.1038/s41564-022-01091-2
WT	*A. fumigatus*	CYP51	Annotated in this study	ERR9791689_2	https://doi.org/10.1038/s41564-022-01091-2
WT	*A. fumigatus*	CYP51	Annotated in this study	ERR9791690	https://doi.org/10.1038/s41564-022-01091-2
WT	*A. fumigatus*	CYP51	Annotated in this study	ERR9791690_2	https://doi.org/10.1038/s41564-022-01091-2
NWT	*A. fumigatus*	CYP51	Annotated in this study	ERR9791688	https://doi.org/10.1038/s41564-022-01091-2
NWT	*A. fumigatus*	CYP51	Annotated in this study	ERR9791688_2	https://doi.org/10.1038/s41564-022-01091-2
WT	*A. fumigatus*	CYP51	Annotated in this study	ERR9791691	https://doi.org/10.1038/s41564-022-01091-2
WT	*A. fumigatus*	CYP51	Annotated in this study	ERR9791691_2	https://doi.org/10.1038/s41564-022-01091-2
NWT	*A. fumigatus*	CYP51	Annotated in this study	ERR9791692	https://doi.org/10.1038/s41564-022-01091-2
NWT	*A. fumigatus*	CYP51	Annotated in this study	ERR9791692_2	https://doi.org/10.1038/s41564-022-01091-2
NWT	*A. fumigatus*	CYP51	Annotated in this study	ERR9791693	https://doi.org/10.1038/s41564-022-01091-2
NWT	*A. fumigatus*	CYP51	Annotated in this study	ERR9791693_2	https://doi.org/10.1038/s41564-022-01091-2
NWT	*A. fumigatus*	CYP51	Annotated in this study	ERR9791694	https://doi.org/10.1038/s41564-022-01091-2
NWT	*A. fumigatus*	CYP51	Annotated in this study	ERR9791694_2	https://doi.org/10.1038/s41564-022-01091-2
NWT	*A. fumigatus*	CYP51	Annotated in this study	ERR9791695	https://doi.org/10.1038/s41564-022-01091-2
NWT	*A. fumigatus*	CYP51	Annotated in this study	ERR9791695_2	https://doi.org/10.1038/s41564-022-01091-2
NWT	*A. fumigatus*	CYP51	Annotated in this study	ERR9791696	https://doi.org/10.1038/s41564-022-01091-2
NWT	*A. fumigatus*	CYP51	Annotated in this study	ERR9791696_2	https://doi.org/10.1038/s41564-022-01091-2
WT	*A. fumigatus*	CYP51	Annotated in this study	ERR9791698	https://doi.org/10.1038/s41564-022-01091-2

WT	*A. fumigatus*	CYP51	Annotated in this study	ERR9791698_2	https://doi.org/10.1038/s41564-022-01091-2
NWT	*A. fumigatus*	CYP51	Annotated in this study	ERR9791697	https://doi.org/10.1038/s41564-022-01091-2
NWT	*A. fumigatus*	CYP51	Annotated in this study	ERR9791697_2	https://doi.org/10.1038/s41564-022-01091-2
WT	*A. fumigatus*	CYP51	Annotated in this study	ERR9791699	https://doi.org/10.1038/s41564-022-01091-2
WT	*A. fumigatus*	CYP51	Annotated in this study	ERR9791699_2	https://doi.org/10.1038/s41564-022-01091-2
NWT	*A. fumigatus*	CYP51	Annotated in this study	ERR9791700	https://doi.org/10.1038/s41564-022-01091-2
NWT	*A. fumigatus*	CYP51	Annotated in this study	ERR9791700_2	https://doi.org/10.1038/s41564-022-01091-2
WT	*A. fumigatus*	CYP51	Annotated in this study	ERR9791701	https://doi.org/10.1038/s41564-022-01091-2
WT	*A. fumigatus*	CYP51	Annotated in this study	ERR9791701_2	https://doi.org/10.1038/s41564-022-01091-2
NWT	*A. fumigatus*	CYP51	Annotated in this study	ERR9791702	https://doi.org/10.1038/s41564-022-01091-2
NWT	*A. fumigatus*	CYP51	Annotated in this study	ERR9791702_2	https://doi.org/10.1038/s41564-022-01091-2
NWT	*A. fumigatus*	CYP51	Annotated in this study	ERR9791703	https://doi.org/10.1038/s41564-022-01091-2
NWT	*A. fumigatus*	CYP51	Annotated in this study	ERR9791703_2	https://doi.org/10.1038/s41564-022-01091-2
NWT	*A. fumigatus*	CYP51	Annotated in this study	ERR9791704	https://doi.org/10.1038/s41564-022-01091-2
NWT	*A. fumigatus*	CYP51	Annotated in this study	ERR9791704_2	https://doi.org/10.1038/s41564-022-01091-2
NWT	*A. fumigatus*	CYP51	Annotated in this study	ERR9791705	https://doi.org/10.1038/s41564-022-01091-2
NWT	*A. fumigatus*	CYP51	Annotated in this study	ERR9791705_2	https://doi.org/10.1038/s41564-022-01091-2
NWT	*A. fumigatus*	CYP51	Annotated in this study	ERR9791706	https://doi.org/10.1038/s41564-022-01091-2
NWT	*A. fumigatus*	CYP51	Annotated in this study	ERR9791706_2	https://doi.org/10.1038/s41564-022-01091-2
NWT	*A. fumigatus*	CYP51	Annotated in this study	ERR9791707	https://doi.org/10.1038/s41564-022-01091-2
NWT	*A. fumigatus*	CYP51	Annotated in this study	ERR9791707_2	https://doi.org/10.1038/s41564-022-01091-2
WT	*A. fumigatus*	CYP51	Annotated in this study	ERR9791708	https://doi.org/10.1038/s41564-022-01091-2
WT	*A. fumigatus*	CYP51	Annotated in this study	ERR9791708_2	https://doi.org/10.1038/s41564-022-01091-2
WT	*A. fumigatus*	CYP51	Annotated in this study	ERR9791709	https://doi.org/10.1038/s41564-022-01091-2
WT	*A. fumigatus*	CYP51	Annotated in this study	ERR9791709_2	https://doi.org/10.1038/s41564-022-01091-2
WT	*A. fumigatus*	CYP51	Annotated in this study	ERR9791710	https://doi.org/10.1038/s41564-022-01091-2
WT	*A. fumigatus*	CYP51	Annotated in this study	ERR9791710_2	https://doi.org/10.1038/s41564-022-01091-2
NWT	*A. fumigatus*	CYP51	Annotated in this study	ERR9791711	https://doi.org/10.1038/s41564-022-01091-2
NWT	*A. fumigatus*	CYP51	Annotated in this study	ERR9791711_2	https://doi.org/10.1038/s41564-022-01091-2
NWT	*A. fumigatus*	CYP51	Annotated in this study	ERR9791712	https://doi.org/10.1038/s41564-022-01091-2
NWT	*A. fumigatus*	CYP51	Annotated in this study	ERR9791712_2	https://doi.org/10.1038/s41564-022-01091-2
NWT	*A. fumigatus*	CYP51	Annotated in this study	ERR9791713	https://doi.org/10.1038/s41564-022-01091-2
NWT	*A. fumigatus*	CYP51	Annotated in this study	ERR9791713_2	https://doi.org/10.1038/s41564-022-01091-2
WT	*A. fumigatus*	CYP51	Annotated in this study	ERR9791714	https://doi.org/10.1038/s41564-022-01091-2
WT	*A. fumigatus*	CYP51	Annotated in this study	ERR9791714_2	https://doi.org/10.1038/s41564-022-01091-2
WT	*A. fumigatus*	CYP51	Annotated in this study	ERR9791715	https://doi.org/10.1038/s41564-022-01091-2
WT	*A. fumigatus*	CYP51	Annotated in this study	ERR9791715_2	https://doi.org/10.1038/s41564-022-01091-2
WT	*A. fumigatus*	CYP51	Annotated in this study	ERR9791716	https://doi.org/10.1038/s41564-022-01091-2
WT	*A. fumigatus*	CYP51	Annotated in this study	ERR9791716_2	https://doi.org/10.1038/s41564-022-01091-2
NWT	*A. fumigatus*	CYP51	Annotated in this study	ERR9791717	https://doi.org/10.1038/s41564-022-01091-2
NWT	*A. fumigatus*	CYP51	Annotated in this study	ERR9791717_2	https://doi.org/10.1038/s41564-022-01091-2
NWT	*A. fumigatus*	CYP51	Annotated in this study	ERR9791718	https://doi.org/10.1038/s41564-022-01091-2
NWT	*A. fumigatus*	CYP51	Annotated in this study	ERR9791718_2	https://doi.org/10.1038/s41564-022-01091-2
NWT	*A. fumigatus*	CYP51	Annotated in this study	ERR9791719	https://doi.org/10.1038/s41564-022-01091-2
NWT	*A. fumigatus*	CYP51	Annotated in this study	ERR9791719_2	https://doi.org/10.1038/s41564-022-01091-2
NWT	*A. fumigatus*	CYP51	Annotated in this study	ERR9791720	https://doi.org/10.1038/s41564-022-01091-2
NWT	*A. fumigatus*	CYP51	Annotated in this study	ERR9791720_2	https://doi.org/10.1038/s41564-022-01091-2
NWT	*A. fumigatus*	CYP51	Annotated in this study	ERR9791721	https://doi.org/10.1038/s41564-022-01091-2
NWT	*A. fumigatus*	CYP51	Annotated in this study	ERR9791721_2	https://doi.org/10.1038/s41564-022-01091-2

NWT	*A. fumigatus*	CYP51	Annotated in this study	ERR9791722	https://doi.org/10.1038/s41564-022-01091-2
NWT	*A. fumigatus*	CYP51	Annotated in this study	ERR9791722_2	https://doi.org/10.1038/s41564-022-01091-2
NWT	*A. fumigatus*	CYP51	Annotated in this study	ERR9791723	https://doi.org/10.1038/s41564-022-01091-2
NWT	*A. fumigatus*	CYP51	Annotated in this study	ERR9791723_2	https://doi.org/10.1038/s41564-022-01091-2
WT	*A. fumigatus*	CYP51	Annotated in this study	ERR9791724	https://doi.org/10.1038/s41564-022-01091-2
WT	*A. fumigatus*	CYP51	Annotated in this study	ERR9791724_2	https://doi.org/10.1038/s41564-022-01091-2
NWT	*A. fumigatus*	CYP51	Annotated in this study	ERR9791725	https://doi.org/10.1038/s41564-022-01091-2
NWT	*A. fumigatus*	CYP51	Annotated in this study	ERR9791725_2	https://doi.org/10.1038/s41564-022-01091-2
WT	*A. fumigatus*	CYP51	Annotated in this study	ERR9791726	https://doi.org/10.1038/s41564-022-01091-2
WT	*A. fumigatus*	CYP51	Annotated in this study	ERR9791726_2	https://doi.org/10.1038/s41564-022-01091-2
WT	*A. fumigatus*	CYP51	Annotated in this study	ERR9791727	https://doi.org/10.1038/s41564-022-01091-2
WT	*A. fumigatus*	CYP51	Annotated in this study	ERR9791727_2	https://doi.org/10.1038/s41564-022-01091-2
WT	*A. fumigatus*	CYP51	Annotated in this study	ERR9791728	https://doi.org/10.1038/s41564-022-01091-2
WT	*A. fumigatus*	CYP51	Annotated in this study	ERR9791728_2	https://doi.org/10.1038/s41564-022-01091-2
WT	*A. fumigatus*	CYP51	Annotated in this study	ERR9791729	https://doi.org/10.1038/s41564-022-01091-2
WT	*A. fumigatus*	CYP51	Annotated in this study	ERR9791729_2	https://doi.org/10.1038/s41564-022-01091-2
WT	*A. fumigatus*	CYP51	Annotated in this study	ERR9791730	https://doi.org/10.1038/s41564-022-01091-2
WT	*A. fumigatus*	CYP51	Annotated in this study	ERR9791730_2	https://doi.org/10.1038/s41564-022-01091-2
WT	*A. fumigatus*	CYP51	Annotated in this study	ERR9791731	https://doi.org/10.1038/s41564-022-01091-2
WT	*A. fumigatus*	CYP51	Annotated in this study	ERR9791731_2	https://doi.org/10.1038/s41564-022-01091-2
WT	*A. fumigatus*	CYP51	Annotated in this study	ERR9791732	https://doi.org/10.1038/s41564-022-01091-2
WT	*A. fumigatus*	CYP51	Annotated in this study	ERR9791732_2	https://doi.org/10.1038/s41564-022-01091-2
NWT	*A. fumigatus*	CYP51	Annotated in this study	ERR9791733	https://doi.org/10.1038/s41564-022-01091-2
NWT	*A. fumigatus*	CYP51	Annotated in this study	ERR9791733_2	https://doi.org/10.1038/s41564-022-01091-2
WT	*A. fumigatus*	CYP51	Annotated in this study	ERR9791734	https://doi.org/10.1038/s41564-022-01091-2
WT	*A. fumigatus*	CYP51	Annotated in this study	ERR9791734_2	https://doi.org/10.1038/s41564-022-01091-2
WT	*A. fumigatus*	CYP51	Annotated in this study	ERR9791735	https://doi.org/10.1038/s41564-022-01091-2
WT	*A. fumigatus*	CYP51	Annotated in this study	ERR9791735_2	https://doi.org/10.1038/s41564-022-01091-2
WT	*A. fumigatus*	CYP51	Annotated in this study	ERR9791736	https://doi.org/10.1038/s41564-022-01091-2
WT	*A. fumigatus*	CYP51	Annotated in this study	ERR9791736_2	https://doi.org/10.1038/s41564-022-01091-2
WT	*A. fumigatus*	CYP51	Annotated in this study	ERR9791738	https://doi.org/10.1038/s41564-022-01091-2
WT	*A. fumigatus*	CYP51	Annotated in this study	ERR9791738_2	https://doi.org/10.1038/s41564-022-01091-2
WT	*A. fumigatus*	CYP51	Annotated in this study	ERR9791739	https://doi.org/10.1038/s41564-022-01091-2
WT	*A. fumigatus*	CYP51	Annotated in this study	ERR9791739_2	https://doi.org/10.1038/s41564-022-01091-2
WT	*A. fumigatus*	CYP51	Annotated in this study	ERR9791737	https://doi.org/10.1038/s41564-022-01091-2
WT	*A. fumigatus*	CYP51	Annotated in this study	ERR9791737_2	https://doi.org/10.1038/s41564-022-01091-2
WT	*A. fumigatus*	CYP51	Annotated in this study	ERR9791740	https://doi.org/10.1038/s41564-022-01091-2
WT	*A. fumigatus*	CYP51	Annotated in this study	ERR9791740_2	https://doi.org/10.1038/s41564-022-01091-2
WT	*A. fumigatus*	CYP51	Annotated in this study	ERR9791744	https://doi.org/10.1038/s41564-022-01091-2
WT	*A. fumigatus*	CYP51	Annotated in this study	ERR9791744_2	https://doi.org/10.1038/s41564-022-01091-2
WT	*A. fumigatus*	CYP51	Annotated in this study	ERR9791741	https://doi.org/10.1038/s41564-022-01091-2
WT	*A. fumigatus*	CYP51	Annotated in this study	ERR9791741_2	https://doi.org/10.1038/s41564-022-01091-2
WT	*A. fumigatus*	CYP51	Annotated in this study	ERR9791745	https://doi.org/10.1038/s41564-022-01091-2
WT	*A. fumigatus*	CYP51	Annotated in this study	ERR9791745_2	https://doi.org/10.1038/s41564-022-01091-2
WT	*A. fumigatus*	CYP51	Annotated in this study	ERR9791748	https://doi.org/10.1038/s41564-022-01091-2
WT	*A. fumigatus*	CYP51	Annotated in this study	ERR9791748_2	https://doi.org/10.1038/s41564-022-01091-2
WT	*A. fumigatus*	CYP51	Annotated in this study	ERR9791743	https://doi.org/10.1038/s41564-022-01091-2
WT	*A. fumigatus*	CYP51	Annotated in this study	ERR9791743_2	https://doi.org/10.1038/s41564-022-01091-2
WT	*A. fumigatus*	CYP51	Annotated in this study	ERR9791746	https://doi.org/10.1038/s41564-022-01091-2

WT	*A. fumigatus*	CYP51	Annotated in this study	ERR9791746_2	https://doi.org/10.1038/s41564-022-01091-2
WT	*A. fumigatus*	CYP51	Annotated in this study	ERR9791749	https://doi.org/10.1038/s41564-022-01091-2
WT	*A. fumigatus*	CYP51	Annotated in this study	ERR9791749_2	https://doi.org/10.1038/s41564-022-01091-2
WT	*A. fumigatus*	CYP51	Annotated in this study	ERR9791747	https://doi.org/10.1038/s41564-022-01091-2
WT	*A. fumigatus*	CYP51	Annotated in this study	ERR9791747_2	https://doi.org/10.1038/s41564-022-01091-2
NWT	*A. fumigatus*	CYP51	Annotated in this study	ERR9791751	https://doi.org/10.1038/s41564-022-01091-2
NWT	*A. fumigatus*	CYP51	Annotated in this study	ERR9791751_2	https://doi.org/10.1038/s41564-022-01091-2
WT	*A. fumigatus*	CYP51	Annotated in this study	ERR9791750	https://doi.org/10.1038/s41564-022-01091-2
WT	*A. fumigatus*	CYP51	Annotated in this study	ERR9791750_2	https://doi.org/10.1038/s41564-022-01091-2
WT	*A. fumigatus*	CYP51	Annotated in this study	ERR9791752	https://doi.org/10.1038/s41564-022-01091-2
WT	*A. fumigatus*	CYP51	Annotated in this study	ERR9791752_2	https://doi.org/10.1038/s41564-022-01091-2
WT	*A. fumigatus*	CYP51	Annotated in this study	ERR9791753	https://doi.org/10.1038/s41564-022-01091-2
WT	*A. fumigatus*	CYP51	Annotated in this study	ERR9791753_2	https://doi.org/10.1038/s41564-022-01091-2
WT	*A. fumigatus*	CYP51	Annotated in this study	ERR9791754	https://doi.org/10.1038/s41564-022-01091-2
WT	*A. fumigatus*	CYP51	Annotated in this study	ERR9791754_2	https://doi.org/10.1038/s41564-022-01091-2
WT	*A. fumigatus*	CYP51	Annotated in this study	ERR9791755	https://doi.org/10.1038/s41564-022-01091-2
WT	*A. fumigatus*	CYP51	Annotated in this study	ERR9791755_2	https://doi.org/10.1038/s41564-022-01091-2
WT	*A. fumigatus*	CYP51	Annotated in this study	ERR9791756	https://doi.org/10.1038/s41564-022-01091-2
WT	*A. fumigatus*	CYP51	Annotated in this study	ERR9791756_2	https://doi.org/10.1038/s41564-022-01091-2
WT	*A. fumigatus*	CYP51	Annotated in this study	ERR9791757	https://doi.org/10.1038/s41564-022-01091-2
WT	*A. fumigatus*	CYP51	Annotated in this study	ERR9791757_2	https://doi.org/10.1038/s41564-022-01091-2
WT	*A. fumigatus*	CYP51	Annotated in this study	ERR9791758	https://doi.org/10.1038/s41564-022-01091-2
WT	*A. fumigatus*	CYP51	Annotated in this study	ERR9791758_2	https://doi.org/10.1038/s41564-022-01091-2
WT	*A. fumigatus*	CYP51	Annotated in this study	ERR9791758_3	https://doi.org/10.1038/s41564-022-01091-2
WT	*A. fumigatus*	CYP51	Annotated in this study	ERR9791759	https://doi.org/10.1038/s41564-022-01091-2
WT	*A. fumigatus*	CYP51	Annotated in this study	ERR9791759_2	https://doi.org/10.1038/s41564-022-01091-2
WT	*A. fumigatus*	CYP51	Annotated in this study	ERR9791760	https://doi.org/10.1038/s41564-022-01091-2
WT	*A. fumigatus*	CYP51	Annotated in this study	ERR9791760_2	https://doi.org/10.1038/s41564-022-01091-2
NWT	*A. fumigatus*	CYP51	Annotated in this study	ERR9791761	https://doi.org/10.1038/s41564-022-01091-2
NWT	*A. fumigatus*	CYP51	Annotated in this study	ERR9791761_2	https://doi.org/10.1038/s41564-022-01091-2
NWT	*A. fumigatus*	CYP51	Annotated in this study	ERR9791762	https://doi.org/10.1038/s41564-022-01091-2
NWT	*A. fumigatus*	CYP51	Annotated in this study	ERR9791762_2	https://doi.org/10.1038/s41564-022-01091-2
WT	*A. fumigatus*	CYP51	Annotated in this study	ERR9791763	https://doi.org/10.1038/s41564-022-01091-2
WT	*A. fumigatus*	CYP51	Annotated in this study	ERR9791763_2	https://doi.org/10.1038/s41564-022-01091-2
WT	*A. fumigatus*	CYP51	Annotated in this study	ERR9791765	https://doi.org/10.1038/s41564-022-01091-2
WT	*A. fumigatus*	CYP51	Annotated in this study	ERR9791765_2	https://doi.org/10.1038/s41564-022-01091-2
WT	*A. fumigatus*	CYP51	Annotated in this study	ERR9791764	https://doi.org/10.1038/s41564-022-01091-2
WT	*A. fumigatus*	CYP51	Annotated in this study	ERR9791764_2	https://doi.org/10.1038/s41564-022-01091-2
WT	*A. fumigatus*	CYP51	Annotated in this study	ERR9791766	https://doi.org/10.1038/s41564-022-01091-2
WT	*A. fumigatus*	CYP51	Annotated in this study	ERR9791766_2	https://doi.org/10.1038/s41564-022-01091-2
NWT	*A. fumigatus*	CYP51	Annotated in this study	ERR9791767	https://doi.org/10.1038/s41564-022-01091-2
NWT	*A. fumigatus*	CYP51	Annotated in this study	ERR9791767_2	https://doi.org/10.1038/s41564-022-01091-2
NWT	*A. fumigatus*	CYP51	Annotated in this study	ERR9791768	https://doi.org/10.1038/s41564-022-01091-2
NWT	*A. fumigatus*	CYP51	Annotated in this study	ERR9791768_2	https://doi.org/10.1038/s41564-022-01091-2
NWT	*A. fumigatus*	CYP51	Annotated in this study	ERR9791769	https://doi.org/10.1038/s41564-022-01091-2
NWT	*A. fumigatus*	CYP51	Annotated in this study	ERR9791769_2	https://doi.org/10.1038/s41564-022-01091-2
NWT	*A. fumigatus*	CYP51	Annotated in this study	ERR9791770	https://doi.org/10.1038/s41564-022-01091-2
NWT	*A. fumigatus*	CYP51	Annotated in this study	ERR9791770_2	https://doi.org/10.1038/s41564-022-01091-2
NWT	*A. fumigatus*	CYP51	Annotated in this study	ERR9791771	https://doi.org/10.1038/s41564-022-01091-2

NWT	*A. fumigatus*	CYP51	Annotated in this study		ERR9791771_2	https://doi.org/10.1038/s41564-022-01091-2
NWT	*A. fumigatus*	CYP51	Annotated in this study		ERR9791772	https://doi.org/10.1038/s41564-022-01091-2
NWT	*A. fumigatus*	CYP51	Annotated in this study		ERR9791772_2	https://doi.org/10.1038/s41564-022-01091-2
NWT	*A. fumigatus*	CYP51	Annotated in this study		ERR9791773	https://doi.org/10.1038/s41564-022-01091-2
NWT	*A. fumigatus*	CYP51	Annotated in this study		ERR9791773_2	https://doi.org/10.1038/s41564-022-01091-2
WT	*A. fumigatus*	CYP51	Annotated in this study		ERR9791774	https://doi.org/10.1038/s41564-022-01091-2
WT	*A. fumigatus*	CYP51	Annotated in this study		ERR9791774_2	https://doi.org/10.1038/s41564-022-01091-2
WT	*A. fumigatus*	CYP51	Annotated in this study		ERR9791775	https://doi.org/10.1038/s41564-022-01091-2
WT	*A. fumigatus*	CYP51	Annotated in this study		ERR9791775_2	https://doi.org/10.1038/s41564-022-01091-2
WT	*A. fumigatus*	CYP51	Annotated in this study		ERR9791776	https://doi.org/10.1038/s41564-022-01091-2
WT	*A. fumigatus*	CYP51	Annotated in this study		ERR9791776_2	https://doi.org/10.1038/s41564-022-01091-2
NWT	*A. fumigatus*	CYP51	Annotated in this study		ERR9791777	https://doi.org/10.1038/s41564-022-01091-2
NWT	*A. fumigatus*	CYP51	Annotated in this study		ERR9791777_2	https://doi.org/10.1038/s41564-022-01091-2
NWT	*A. fumigatus*	CYP51	Annotated in this study		ERR9791778	https://doi.org/10.1038/s41564-022-01091-2
NWT	*A. fumigatus*	CYP51	Annotated in this study		ERR9791778_2	https://doi.org/10.1038/s41564-022-01091-2
NWT	*A. fumigatus*	CYP51	Annotated in this study		ERR9791779	https://doi.org/10.1038/s41564-022-01091-2
NWT	*A. fumigatus*	CYP51	Annotated in this study		ERR9791779_2	https://doi.org/10.1038/s41564-022-01091-2
NWT	*A. fumigatus*	CYP51	Annotated in this study		ERR9791782	https://doi.org/10.1038/s41564-022-01091-2
NWT	*A. fumigatus*	CYP51	Annotated in this study		ERR9791782_2	https://doi.org/10.1038/s41564-022-01091-2
NWT	*A. fumigatus*	CYP51	Annotated in this study		ERR9791781	https://doi.org/10.1038/s41564-022-01091-2
NWT	*A. fumigatus*	CYP51	Annotated in this study		ERR9791781_2	https://doi.org/10.1038/s41564-022-01091-2
WT	*A. fumigatus*	CYP51	Annotated in this study		ERR9791780	https://doi.org/10.1038/s41564-022-01091-2
WT	*A. fumigatus*	CYP51	Annotated in this study		ERR9791780_2	https://doi.org/10.1038/s41564-022-01091-2
